# Revealing interactions of layered polymeric materials at solid-liquid interface for building solvent compatibility charts for 3D printing applications

**DOI:** 10.1038/s41598-019-56350-w

**Published:** 2019-12-27

**Authors:** Kirill S. Erokhin, Evgeniy G. Gordeev, Valentine P. Ananikov

**Affiliations:** Zelinsky Institute of Organic Chemistry, Russian Academy of Sciences, Leninsky prospekt 47, Moscow, 119991 Russia

**Keywords:** Chemical engineering, Chemical engineering

## Abstract

Poor stability of 3D printed plastic objects in a number of solvents limits several important applications in engineering, chemistry and biology. Due to layered type of assembling, 3D-printed surfaces possess rather different properties as compared to bulk surfaces made by other methods. Here we study fundamental interactions at the solid-liquid interface and evaluate polymeric materials towards advanced additive manufacturing. A simple and universal stability test was developed for 3D printed parts and applied to a variety of thermoplastics. Specific modes of resistance/destruction were described for different plastics and their compatibility to a representative scope of solvents (aqueous and organic) was evaluated. Classification and characterization of destruction modes for a wide range of conditions (including geometry and 3D printing parameters) were carried out. Key factors of tolerance to solvent media were investigated by electron microscopy. We show that the overall stability and the mode of destruction depend on chemical properties of the polymer and the nature of interactions at the solid-liquid interface. Importantly, stability also depends on the layered microstructure of the sample, which is defined by 3D printing parameters. Developed solvent compatibility charts for a wide range of polymeric materials (ABS, PLA, PLA-Cu, PETG, SBS, Ceramo, HIPS, Primalloy, Photoresin, Nylon, Nylon-C, POM, PE, PP) and solvents represent an important benchmark for practical applications.

## Introduction

Additive manufacturing technologies become increasingly popular in chemical engineering, digital prototyping, microreactor design, and healthcare device development, among many other areas. Nowadays, 3D printing is actively involved in several areas of materials research and development^1–4^. It allows to produce complex parts, manufacturing of which by conventional methods would be time-consuming and expensive; its increasing prevalence is associated with the launch of inexpensive desktop 3D printers. Non-waste, versatile and inexpensive, the additive manufacturing technologies allow rapid and accurate production of prototypes and functional parts. The advent of 3D printing is sometimes referred to as a new industrial revolution^5^. In basic and applied research, 3D printing has already been adapted for the needs of biotechnology^6–9^, energetics^10,11^, optics^12,13^, novel engineering applications^14–16^, pharmaceutics^17^ and medicine^18–21^, as well as the development of separation devices^22^, sensors^23^, and microreactors^24^. It has also proved itself in chemistry, particularly in fine organic synthesis^25,26^, analytical chemistry^27^, catalytic system design^28,29^, and flow chemistry^30–32^.

Additive manufacturing technologies include several methods, which differ by layer formation principles and scopes of suitable materials. A highly popular additive manufacturing technology, called Fused Deposition Modeling (FDM), is the layer-by-layer deposition of thermoplastic polymer by extrusion via heated nozzle. Its main advantages are the low cost of personal FDM 3D printers^33^ and the wide assortment of suitable thermoplastics^34,35^. The FDM principle allows using certain non-conventional materials, e.g. some biopolymers^36,37^, polymer composites^38,39^, metal oxides^40^ or glass^41^. It offers the widest possibilities of 3D printing for various conceivable purposes.

Current challenges in 3D printing include not only prototyping, but also building functional parts for practical applications. In chemistry, 3D printing has mediated important innovations in design of the reactors for multistep organic synthesis of pharmaceuticals^42^. In medicine, it affords made-to-measure prostheses and implants at relatively low costs, while 3D bioprinting with living tissues allows creating artificial organs for autologous transplantations. Agricultural applications of 3D printing include minifarms and 3D-ponics^43^. Such extensive use of 3D printed products makes it important to select appropriate materials, in particular, resistant to various organic and inorganic substances. The choice of materials will always be application-determining for applying in chemical industry, biotechnology, engineering, catalysis, and basic research. Despite the evidence on stability of raw thermoplastics in different media, stability of FDM parts made of these plastics is still an open question. Melting of the filament during the procedure can substantially modify its microstructure; the extent and character of this modification may depend on printing parameters. Besides, the FDM parts are highly porous^44^. These factors significantly affect stability of FDM parts in different media, as compared with parts made of the same materials by classical approaches (molding, turning, stamping etc.). Plastic functional parts of equipment may be in contact with water or alcohols^45,46^; in some cases they are exposed to organic solvents^47^.

Several known thermoplastic polymers show high chemical and thermal resistance in combination with exceptional mechanical strength. These so-called superconstruction materials include polyether ether ketone (PEEK), polyphenylsulfone (PPSU) and polyetherimide (Ultem)^48,49^. However, FDM with superconstruction materials requires high-temperature extruders (350–400 °C), as well as the constantly high temperature within printing chamber; these conditions would require utilization of specifically upgraded and more expensive 3D printers. Besides, the materials themselves are expensive (as compared with the conventional production-grade thermoplastics), which makes their routine use not very common. Despite the high demand for strong and chemically resistant FDM materials with low shrinkage, the efforts towards their development are still limited.

As an important limitation, 3D printing is poorly suitable for production of plastic objects to be exposed to liquid media. The limitation is due to susceptibility of the plastics to the action of liquid chemicals. Pilot experiments with commodity thermoplastics disproved the suitability of FDM printed parts for direct contacts with solvents and solutions.

This article describes evaluation of processes at solid-liquid interface and connection of these phenomena with stability of 3D printed polymeric parts. The majority of the studied polymers are production-grade thermoplastics compatible with desktop 3D printers. A number of thermoplastic polymers were comprehensively studied in order to assess their compatibility with different solvents used in chemical technologies (including fine organic synthesis, pharmaceutics and paint industry) and every-day research practice in a lab. We show that chemical resistance of 3D printed parts can be significantly improved by reasonable adjustment of printing parameters. Structurally important micro-scale processes at the solid-liquid interface are studied by electron microscopy.

## Results

### Stability tests for various plastic materials in different solvents

**Figure 1:**
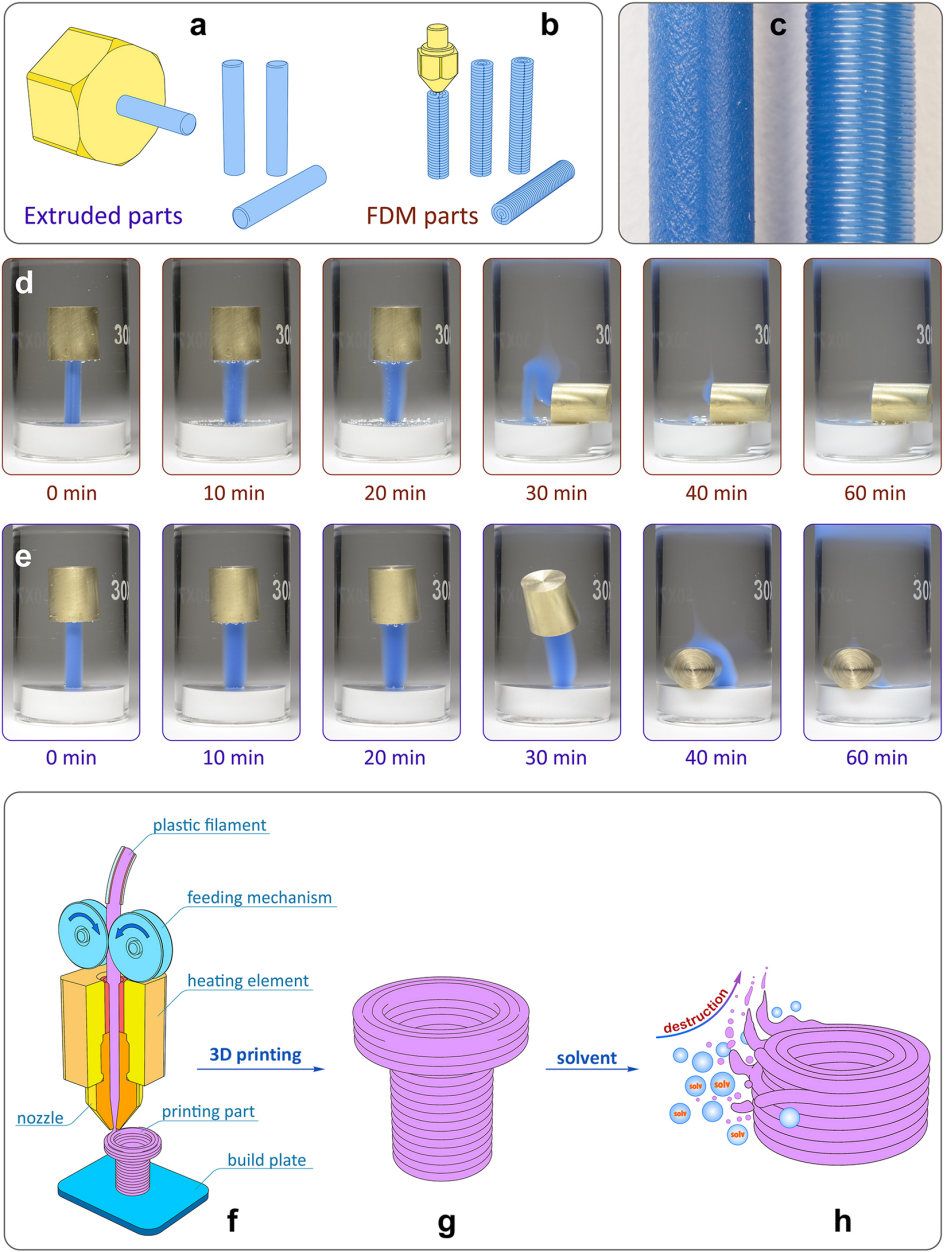
Representation of stability test of FDM parts in liquid media. (**a**) PLA parts made by standard extruding technology; (**b**) PLA parts made in this work by FDM 3D printing; (**c**) macrophotos of the extruded part (left) and FDM printed part (right) with same diameter 2.85 mm; (**d,e**) snapshots of the chemical resistance tests in DCM for the 3D-printed part (extrusion multiplier k = 0.9) and for the extruded part, respectively, with a brass cylinder as an indicator of integrity (Supplementary Movies S1, S2); (**f**) the principle of FDM-based additive manufacturing; (**g**) layered structure produced by FDM, (**h**) destruction of the 3D-printed surface due to interaction with a solvent.

3D printed parts have a specific type of surface and contain various microstructural defects (e.g. pores, spaces and gaps between the layers)^50–52^. Solvent molecules can penetrate into these defects thereby increasing the surface of solid-liquid interface and reducing the resistance to the action of solvents. To assess the impact of specific surface and microstructural defects on the chemical resistance of 3D printed objects, we carried out a comparative model experiment involving extruded cylindrical part (with a diameter of 2.85 mm) and its exact 3D printed copy with the same material, geometry and mass (Fig. 1a–c). Both PLA parts were tested by immersion in DCM. Noticeably, a different behavior was observed and 3D printed part lost its shape faster (Fig. 1d,e). The comparison shows that mode of manufacturing (traditional vs. 3D printing) is highly important for the real performance in a contact with liquid. Additive manufacturing procedure results in specific final structure of the object (Fig. 1b,c,g), thus a different type of interactions at the solid-liquid interface is responsible for maintaining structural integrity and achieving overall stability (Fig. 1h). This test utilized exactly the same material for both samples and demonstrated that manufacturing method is of much importance. Thus, simple solvent compatibility chart (known for bulk polymeric materials) is not applicable for 3D-printed items made by FDM and a dedicated assessment is required.

A special procedure was optimized for rapid assessment of various plastic materials in 3D printed objects. Identical model parts for studying chemical resistance (a hollow cylindrical part with a 10 mm diameter of bottom and a total height of 17 mm (Fig. 1g) were built from wide range of FDM plastics. In this article widely known thermoplastics (PLA, ABS, SBS, HIPS, PETG, Nylon, POM, PP, and PE) were analyzed as well as novel ones developed especially for FDM printing: PLA-Cu (PLA with a filler of finely ground copper), Primalloy, Ceramo, Nylon-C (Nylon with a filler of carbon fibers). The part made of a stereolithography grade photoresin by photopolymerization was also tested (Supplementary Fig. S1).

During the test procedure, the printed parts were placed individually into empty vessels and topped with steel or glass beads to indicate the destruction. Subsequent addition of a solvent was followed by snapshots of the sample at multiple time points in the course of 20 hours. Twelve different solvents were used in the experiments: dichloromethane (DCM) (Supplementary Figs. S1–S8), tetrahydrofurane (THF) (Supplementary Figs. S9–S11), acetone (Supplementary Figs. S12–S14), dimethylformamide (DMF) (Supplementary Figs. S15–S17), toluene (Supplementary Figs. S18–S21), ethyl acetate (Supplementary Figs. S22–S25), triethylamine (TEA) (Supplementary Figs. S26–S28), acetic acid (Supplementary Figs. S29–S31), ethanol (Supplementary Figs. S32–S34), 0.5 M aqueous solution of sulfuric acid (Supplementary Figs. S35–S37), 1 M aqueous solution of sodium hydroxide (Supplementary Figs. S38–S40), and water (Supplementary Figs. S41–S43). Continuous presence of the bead provided convenient indication of stability/destruction that would otherwise be difficult to record. Without the bead, the loss of the mechanical strength would be difficult to notice; the presence of the bead made the loss of structural integrity clearly observable. Since FDM printed parts are hollow cylinders, bead contacts with the part along the perimeter regularly distributing a load (Supplementary Fig. S51).

**Figure 2:**
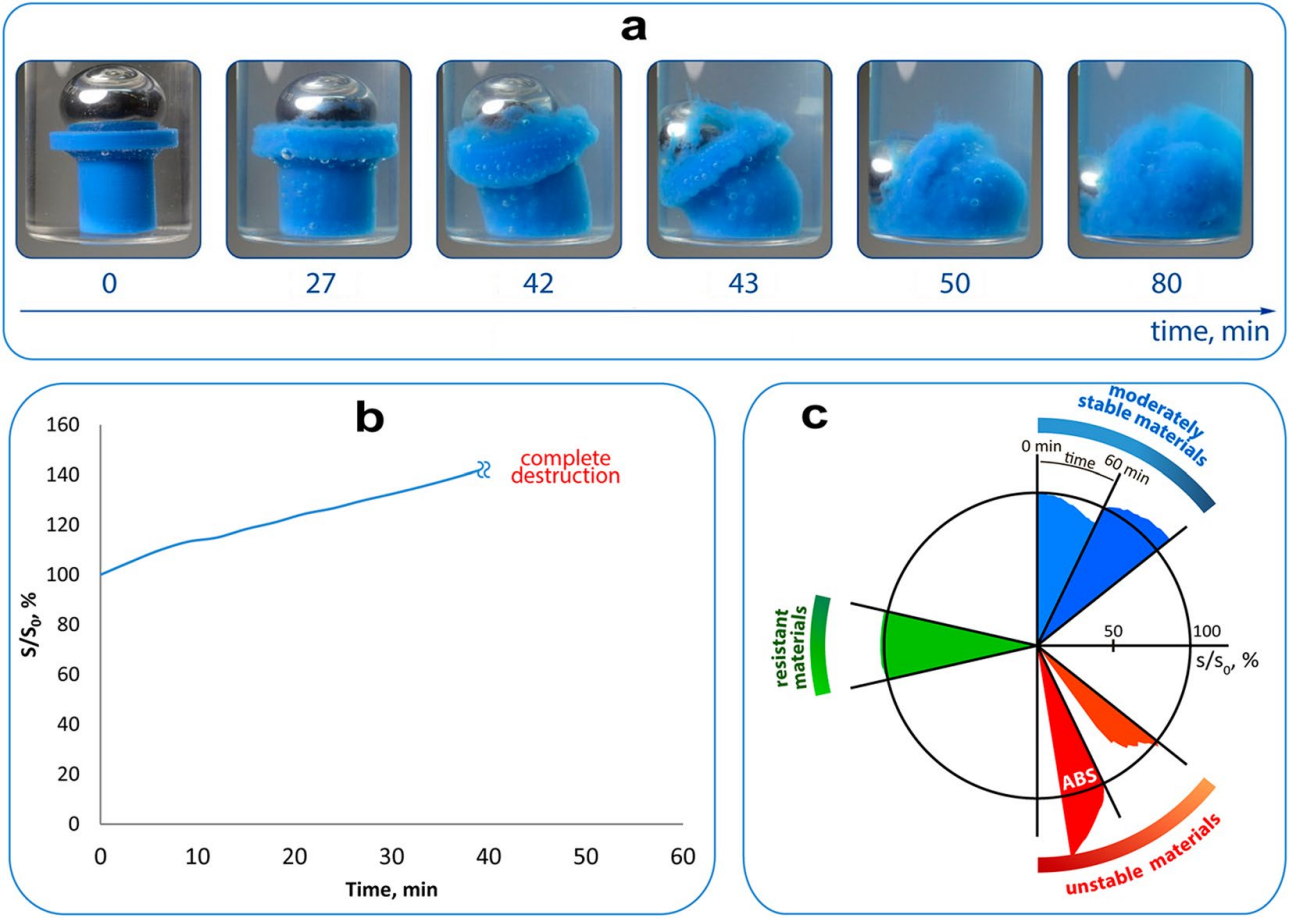
An example of destruction of ABS part in DCM with a metal bead as an indicator of integrity. (**a**) A series of snapshots; (**b**) corresponding curve reflecting increased visible area occupied by 3D-printed blue cylinder due to destruction process (horizontal axis shows experimental time, vertical axis shows actual-to-initial areas ratio); (**c**) examples of representation in a circular diagram: ABS as an unstable material (red) as well as general notations of more stable materials (blue and green).

A representative snapshot series for ABS/DCM system is shown in Fig. 2a. The area covered by the plastic object in an image was used as a quantitative index. An increase in this area during the experiment is indicative of swelling or delamination processes, while the decrease indicates dissolution of the tested object. Destruction curves illustrating changes of the FDM part area in snapshot images have been built; an example is shown in Fig. 2b. Behavior of the curve during the experiment allows to evaluate the resistance for different types of FDM material, while the character of changes indicates the type of destruction. Built curve was placed on circular diagram with the time of the experiment expressed by the angle and the corresponding size ratio expressed by the radial distance (Fig. 2c).

**Figure 3:**
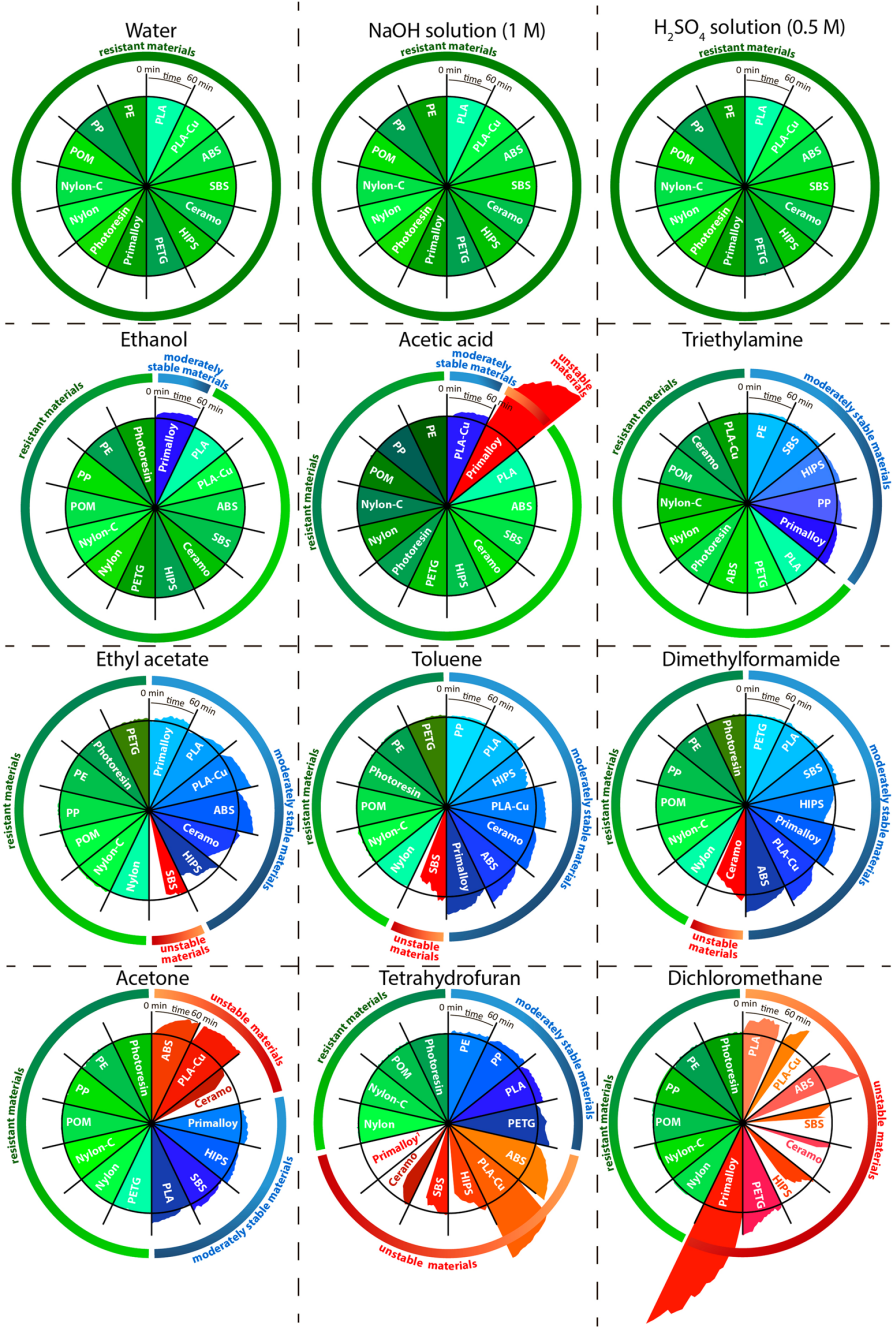
Circular diagrams of change in the FDM part area during 1 h exposure to organic and inorganic solvents. () the material collapses during the experiment (ΔS > 20%): the object loses its shape by dissolution, disintegration and/or delamination; () the material shows moderate stability during the experiment (ΔS = 2–20%), with minor swelling or dissolution of the outer layers, but satisfactory retention of the shape; () the material is stable during the experiment (ΔS < 2%): the object retains its shape, and no dissolution of the outer layers is observed (see Supplementary Movie S3). ^1^Destruction of Primalloy in THF took just a few seconds.

A series of other polymeric materials were evaluated using this simple and efficient approach. The data summarized in Fig. 3 indicate that all tested materials are chemically resistant to water, acidic and basic aqueous solutions, and ethanol (see also Supplementary Table S2). The highest chemical resistance to organic solvents was shown by PP, PE, POM, Nylon, and Nylon-C, whereas the typical FDM materials exemplified by PLA, ABS, SBS, and HIPS showed lower resistance to organic solvents (Fig. 3). PP and PE undergo slight swelling in triethylamine and THF. The resistance of PLA-Cu was accordingly low, with dissolution of the PLA matrix and release of the copper particles in several solvents, and a similar behavior was shown by Ceramo (Supplementary Fig. S4). The only material with elastomer properties, Primalloy, showed moderate resistance to acetone, ethyl acetate and toluene. It is noteworthy that, by contrast with other thermoplastics, Primalloy increases in volume, but retains its shape during swelling (Supplementary Fig. S20). PETG showed high resistance to acetone and toluene; it was also resistant to ethyl acetate and DCM at short-term exposures. The photoresin was resistant to the most of the solvents except DCM, which caused its destruction in the course of 20 hours. Exposure of photoresin to acetic acid, DMF and THF for 20 hours led to swelling of the 3D-printed part.

Despite the observation that many plastics are quite stable in organic solvents for one hour, most of the materials collapse when exposed to the same solvents for 20 hours. In particular, the resistance of FDM products under prolonged exposure to methylene chloride (which is one of the most aggressive organic solvents) is increased in the following series: SBS < PLA-Cu < Ceramo < HIPS < ABS < Primalloy < PLA < PETG < Photoresin <<Nylon, Nylon-C, POM, PE, PP (see Supplementary Table S2 for details).

Two types of beads, indicating the integrity of printed part during the treatment, were used during the study of chemical resistance. A glass bead was used for water and aqueous solutions (to avoid reaction with acids, etc.), whereas a steel bead was used for organic solvents. Because of the mass difference, the beads provided different loads on FDM parts. The influence of this parameter in combination with Archimedean force was examined additionally (Supplementary Fig. S45 and Movie S4). Convincingly enough, in all comparative experiments, the beads of glass and steel dropped down at the same time point. Moreover, in experiments with different levels of the solvent (DCM) in the vessel, FDM parts deteriorated in parallel and collapsed simultaneously. Thus, neither the weight of the bead (with reasonably small load weight), nor the influence of buoyant force may effect the conclusions about relative stability of plastic materials. The shape of the indicator also had no effect on the time of destruction (Supplementary Fig. S46).

Depending on the performance shown in these experiments, the tested materials can be divided into three groups differing by chemical resistance to organic solvents:

1. Susceptible to destruction by the majority of organic solvents (PLA, PLA-Cu, ABS, SBS, Ceramo, Primalloy, and HIPS);
2. Resistant to the majority of organic solvents, but unstable in particular solvents (the photopolymer resin, PETG);
3. Stable at the solid-liquid interface in various organic solvents (PP, PE, POM, Nylon и Nylon-C).

### Mechanistic study of the destruction processes

It is noteworthy that the first group of materials shows different behaviors in aggressive media, depending on both the material and the solvent. The processes at the solid-liquid interfaces have been studied by scanning electron microscopy.

**Figure 4:**
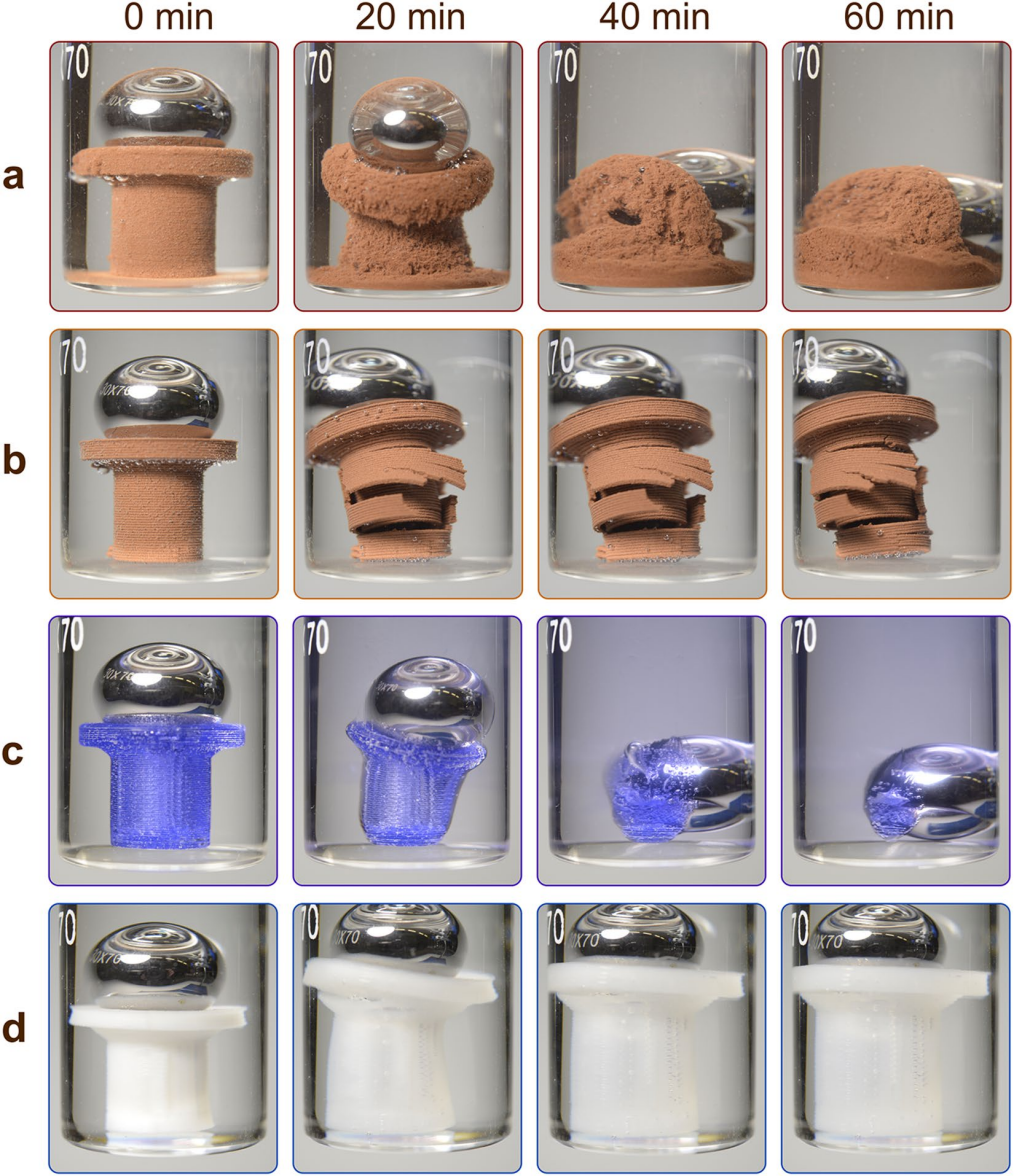
Representative snapshot series of FDM parts during the chemical resistance tests. (**a**) PLA-Cu in DCM – disintegration; (**b**) PLA-Cu in acetone – delamination; (**c**) SBS in DCM – dissolution; (**d**) Primalloy in toluene – swelling (see Supplementary Movie S3).

**Figure 5:**
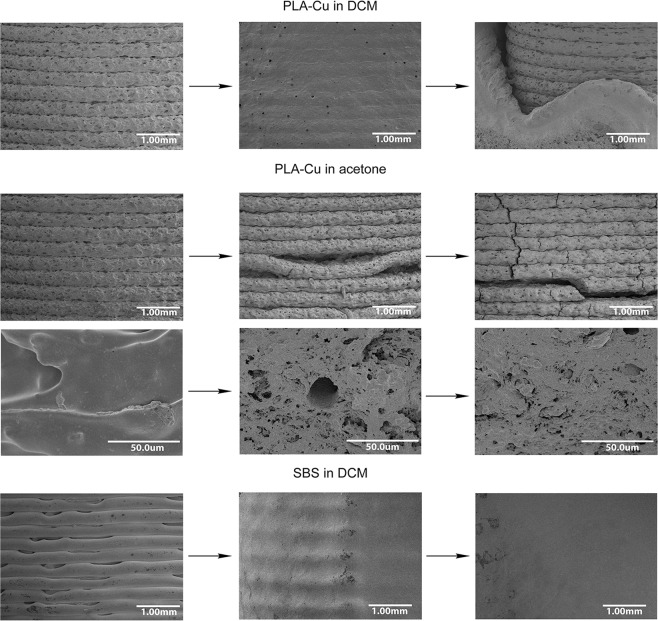
Scanning electron microscopy FE-SEM images of 3D printed surfaces at successive stages of the destruction showing various morphological changes.

The solvent-mediated destruction of filled plastics, e.g. PLA-Cu or Ceramo, is accompanied by gradual release of reinforcing particles along with dissolution of the polymer matrix (Fig. 4a). A more detailed analysis of the destruction process was conducted using field-emission scanning electron microscopy (FE-SEM) (Fig. 5) which allowed analyzing character of destruction of the part most notably between layers. Due to the fact that thickness of the layer is less than 0.5 mm, it is sometimes complicated to clearly define character of destruction using photos made with camera. Before the experiment, the surface of PLA-Cu part shows well-defined layers with pores both within the layers and in between. Treatment with DCM leads to blurring of the layers and gradual development of the surface granularity, which is due to the presence of copper particles. As a result, the outer surface is being washed off. This makes the inner layers susceptible to interaction with the solvent and consecutive dissolution.

Treatment of ABS, PLA and PLA-Cu with acetone leads to gradual segregation of the layers resulting in complete destruction of the part. In the case of ABS, the interlayer adhesion is loosened by swelling and fluxing of the polymer. In contrast, the experiments with PLA and PLA-Cu show no fluxing or swelling (Fig. 4b): the surface of the part cracks not only between the layers, directions of the formed splits are random.

To study the destruction mechanism for PLA and PLA-Cu parts in acetone, the solution left after 20 hours of the PLA-Cu experiment was evaporated, and the weight of dry residue was found to be 0.016 g. Taking into account the initial weight of the part (2 g) and the content of PLA (40%), it can be concluded that destruction of PLA-Cu part is not a sheer consequence of dissolution of the polymer and concomitant reduction of the interlayer adhesion, but represents another type of processes at the solid-liquid interface. It has been shown that some organic solvents (e.g. acetone) promote restructuring of polylactide surface by changing orientation of the pendant methyl-groups at the material’s surface and packing of macromolecule segments^53^. This provides an explanation for cracking of PLA parts in the acetone media. Exposure of a PLA part to the solvent leads to a higher density packing at the part’s surface, which causes a decrease in the surface area and an increase in mechanical strain inside the material. Formation of cracks within the layers is clearly observable in the images (Fig. 5). It should be noted that the surface morphology also changes from smooth to granular.

It is interesting that unfilled PLA in DCM media form the gummy mass, while Cu-filled PLA is dispersed without formation of gummy substance. That is, bonding of FDM parts made of Cu-filled PLA with DCM likely will not to be effective.

In the majority of organic solvents, SBS parts gradually and proportionally decrease in size without swelling or delamination, which is followed by complete visual disappearance of the part and staining of the solution with the dye of the material (Fig. 4c). In microphotographs, the outer surface shows loosening of the layers, with the pores between them. In DCM, blurring of the layers and smoothing of the outer surface (Fig. 5) is followed by dissolution.

All polymer materials are more or less prone to swelling in organic solvents, but Primalloy in DCM swells in the most distinguished manner. Within 1 hour, the total volume of the solvent is absorbed by the swollen gelatinous material, which used to be the FDM part, now filling the entire volume of the glass container. Primalloy also swells in hydrocarbon solvents, e.g. in toluene, but to a much lesser extent and without disintegration (Fig. 4d). Light microscopy assessment of these moderately swollen parts has shown an increase in the size of the fibers and concomitant smoothing of the surface defects (Supplementary Fig. S21); the surface pores disappear because of the increase in packing density. Within 1 h *in vacuo*, the swollen part recovers its original shape with no signs of destruction.

The materials with stable behaviors (PP, POM, Nylon and Nylon-C) were additionally checked for subtle changes in surface microstructure. After 20-hour exposure to DCM, the FDM parts were studied by FE-SEM (Supplementary Figs. S5–S8). POM showed a smooth surface with pores both between and within the layers. The PP surface was textured, with pores between the layers. In the case of Nylon, glib surface and interlayer pores were observed. In contrast, the layers of Nylon-C were textured and had pores both between and within them. No signs of dissolution are observed in the microphotographs, with complete preservation of the surface topography and distinct pores at the layer interfaces.

Destruction mode for a particular object is determined by its microstructure in combination with the type of interaction between the material and the solvent. At the first stage of destruction, the solvent is absorbed by the polymer and penetrates between its chains. The degree of penetration depends on microstructure (surface topography, porosity, etc.) and chemical nature of the polymer. At the next stage, polymer chains undergo partial solvation, which makes them flexible and causes swelling of the object. The degree of swelling is defined by affinity of the polymer to the solvent. For instance, PLA in DCM undergoes swelling that smoothly transits into dissolution. In the cases of limited swelling, the object increases in size without dissolution (e.g. Primalloy parts in toluene).

### The influence of 3D printing parameters on the chemical resistance

The resistance of 3D printed parts to the influence of organic solvents can be improved by means of (1) modification of printing material and (2) optimization of FDM printing parameters. Addition of fillings to the polymer matrix significantly reduces the shrinkage and thus improves the dimensional precision of printing. Fibrous fillers not only reduce the shrinkage but also enhance the mechanical strength of FDM parts and, accordingly, their reliability in composite reactors and other equipment. For example, the unfilled nylon parts shrink significantly, whereas the identical parts printed from the carbon-filled nylon are less prone to shrinkage and also stronger, especially when the force is applied along the layers.

Optimization of FDM parameters and geometry (extrusion multiplier, wall thickness, printing temperature, infill density etc.) allows to reduce microporosity of the printed part and thus enhance its impermeability generally associated with resistance to aggressive media. In previous work^44^, we have shown that extrusion multiplier (k) affects on impermeability and structural integrity of FDM parts. Infill density of course also one of the main parameters which determine stability of FDM parts in solvent media, but in this paper, we studied the stability of thin-walled parts, mainly characteristic for practice of the chemical laboratory. For thin-walled vessels the wall consists of outer and internal perimeters (within FDM printing terminology) and doesn’t contain space for internal infill, i.e. all tested objects in this work are characterized by 100% infill density.

**Figure 6:**
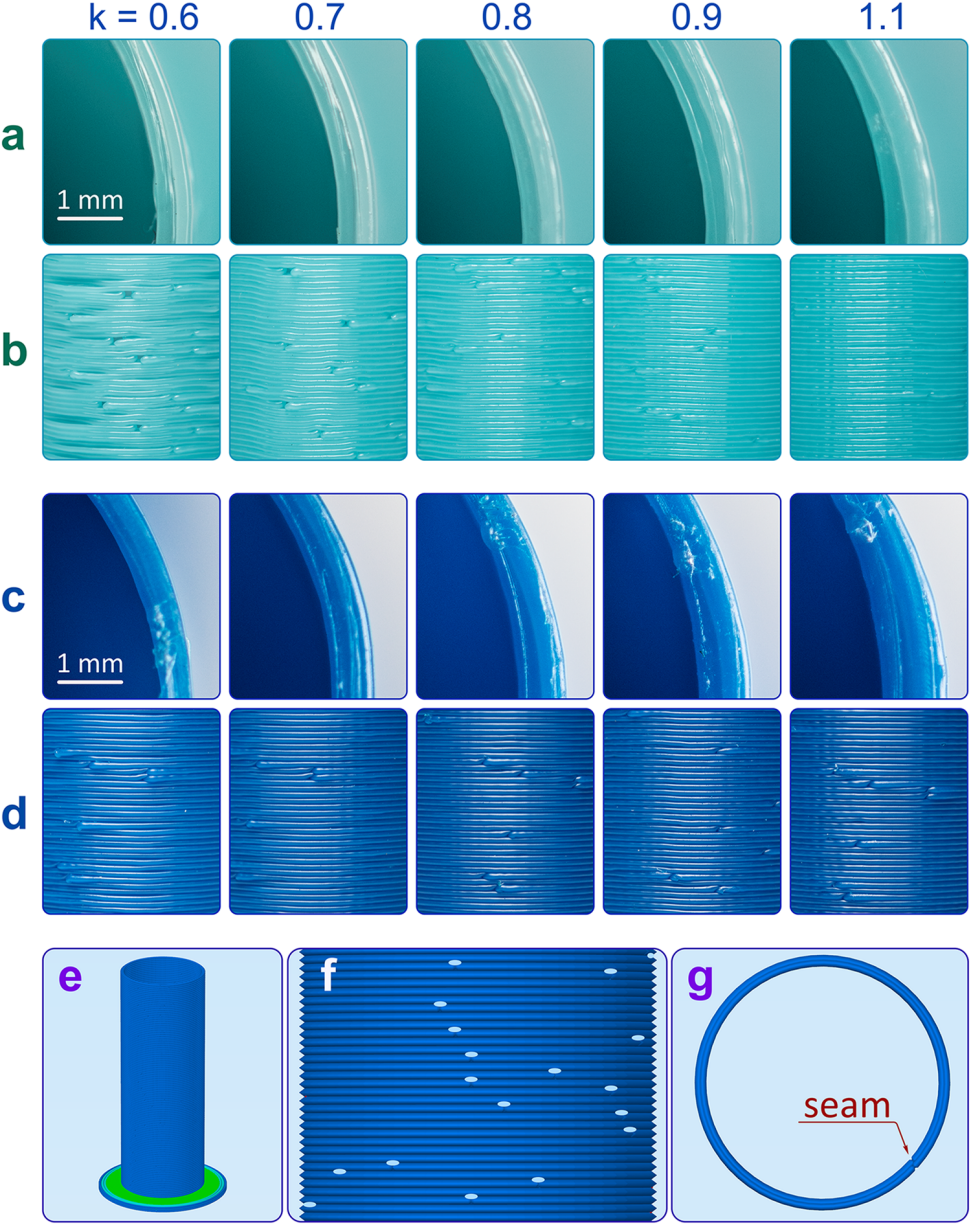
The influence of extrusion multiplier k on structural integrity of FDM parts. (**a**) change in the wall thickness for a cylindrical part made of PLA depending on the extrusion multiplier; (**b**) change in the wall structural integrity for a cylindrical part made of PLA; (**c**) change in the wall thickness for a cylindrical test tube made of ABS; (**d**) change in the wall structural integrity for a cylindrical test tube made of ABS; (**e**) a graphic representation of complete G-code for FDM test tube; (**f**) G-code-defined distribution of the seam points on the FDM test tube wall; (**g**) structure of a single layer of FDM test tube with the seam position denoted by red arrow.

Extrusion multiplier corresponds to the volume flow rate of the molten plastic during FDM 3D printing. To study the effect of k on the stability of FDM parts, we printed a series of thin-walled cylindrical test tubes using different values of k (Fig. 6a–d) within the same G-code (Fig. 6e). At a 0.6 mm wall thickness and a 0.3 mm nozzle diameter, the wall consisted of two concentric perimeters. Each perimeter had a seam (Fig. 6f,g); the seams are one of the main reasons for the porosity of FDM products. To test their chemical resistance, the printed test tubes were filled with dichloromethane. For indication of structural integrity, a brass cylinder was placed on top of each test tube during the experiment.

FDM test tubes manufactured at low values of extrusion multiplier (k = 0.6, 0.7) had prominent defects, most notably through holes in the walls of PLA test tubes (Fig. 6a). ABS test tubes fabricated at low values of extrusion multiplier had such a low interlayer adhesion that even a slight mechanical strain caused delamination. FDM parts printed at higher values of extrusion multiplier (k = 0.9, 1.1) had denser walls. By virtue of the high extrusion multiplier, the defects at the seam points were filled with plastic, which significantly reduced porosity of the wall and increased its stability in liquid media.

Indeed, test tubes fabricated at k = 0.6 exhibited permeability in the experiment: the solvent easily flowed out through the pores and interlayer spaces. As a result, the part was exposed to the solvent not only from the inside but also from the outside, quickly softened and lost its structural integrity during the first minutes of the experiment (Supplementary Figs. S47A and S48A). For k = 0.8, the PLA and ABS test tubes were stable for 5 minutes but began to soften after 10 minutes of interaction with the solvent. The most stable test tubes were obtained at k = 1.1; these tubes retained their structural integrity for 10 minutes. Even after 30 minutes, the PLA test tubes stayed undestroyed, although by this time they noticeably swelled and distended (Supplementary Figs. S47C and S48C). It should be noted that the PLA test tubes were more resistant than the ABS test tubes.

Importantly, all experiments involving a change in the parameter k were carried out by using the same 3D model of a test tube and the same G-code. The increase in k led to a slight increase in the wall thickness and also to a very relevant increase in the wall density. As the enhanced interlayer adhesion stopped the solvent from penetrating into the wall, softening of the plastic proceeded slowly, starting from the surface layers of the wall and only gradually proceeding to the deeper layers; hence the greater stability of FDM parts fabricated at higher extrusion multipliers.

Thus, increase in the parameter k allows printing FDM parts of higher stability at the solid-liquid interfaces. As a proof of principle, resistance to DCM was compared between a series of cylindrical parts, made of PLA by FDM at k = 0.8, 0.9, 1.0, 1.1, 1.2 and 1.3, and a cylindrical part of identical shape manufactured by extrusion (fragment of the conventional filament for FDM printers). FDM parts printed at k = 1.1–1.3 showed the same stability as compared to the extruded one (Supplementary Fig. S50 and Movie S2). It should be noted that the weights of all tested parts were similar (Supplementary Table S3).

## Discussion

On the basis of performed evaluation of 3D printed objects, studied FDM-compatible materials can be divided into three groups:

- The first group includes materials that are stable in gentle aqueous media (PLA, ABS, SBS, HIPS, Primalloy). These materials can be adapted for biotech applications with the non-stringent aqueous media and lenient requirements to the chemical purity of the material (provided it is in compliance with the toxicity standards). PLA has been recently positioned as a promising raw material for biodegradable implants and connecting elements in surgery, and its medical applications are therefore also plausible^54^.
- Materials of the second group (photopolymers and some thermoplastic polymers, e.g. PETG) are resistant to water-miscible organic solvents, first of all, to the low molecular weight alcohols. Such materials may be considered for applications in medicine, especially as they can be sterilized with ethanol or other disinfectants.
- Materials of the third group are resistant to the majority of organic solvents, including the most aggressive DCM, THF and acetone. Excellent stability of such materials allows their application in chemistry for building chemical reactors and fittings, whereas the use of PP and PE in manufacturing of medical tools and consumables has been accepted globally. Of course, this is a basic first-stage assessment only, and more specific testing would be required for a particular application.

**Figure 7:**
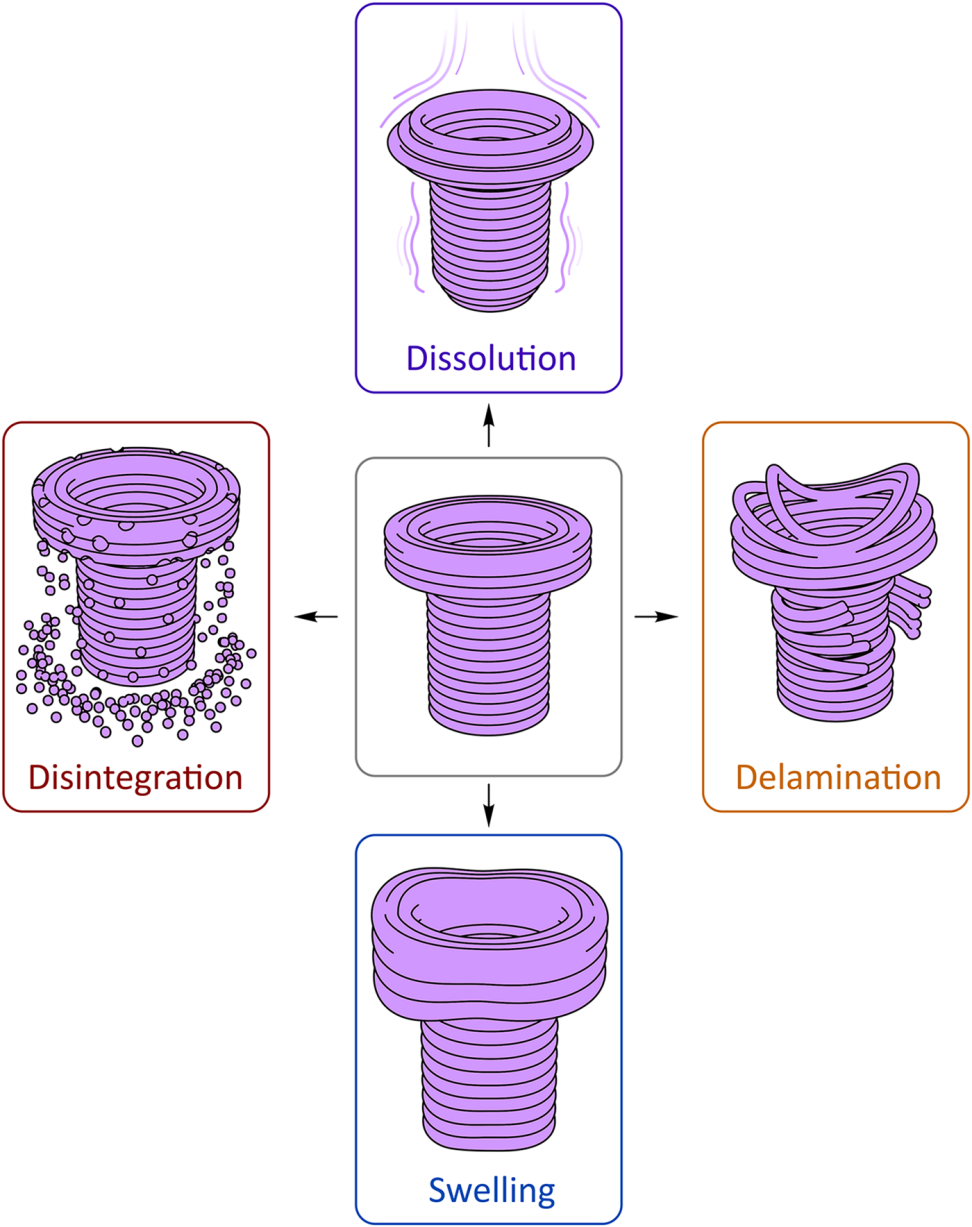
Types of destruction at the solid-liquid interfaces observed for different combinations of polymeric materials and solvents (Supplementary Movie S3).

The observed types of destruction (Fig. 7) can be classified as follows: (1) disintegration; (2) delamination (splitting into separate layers); (3) molecular dissolution; (4) swelling. The simplest type of destruction is dissolution observed for materials with high affinity to solvent. During this process, the polymer chains are solvated and rendered flexible, and gradually transit into the solvent.

Disintegration of materials filled with fine-grained additives consists of dissolution/destruction of the polymer matrix and consecutive release of the insoluble filler particles.

Filling plastic with fine-grained additives fundamentally changes the nature of the destruction of the FDM parts. For example, unfilled PLA under the influence of methylene chloride forms a gluing mass. Therefore, DCM can be used for gluing parts made of PLA. However, PLA filled with copper particles (in such FDM specific material as eCopper aka PLA-Cu in this article) does not form a gluing mass, so DCM will be ineffective for bonding parts made of such material.

Delamination is caused by slight swelling of the layers leading to a decrease in the interlayer adhesion; as a result, the slightly swollen flexible fibers detach from the 3D printed entity. A different character of swelling, with an increase in volume but without losing structural integrity, is typical for elastomers.

The study shows that objects printed by FDM from polyoxymethylene (POM), polyethylene (PE), polypropylene (PP), nylon (Nylon) and carbon-filled nylon (Nylon-C) are resistant to organic solvents. This makes them good candidate materials for various parts of equipment (mixers, separation devices, flow reactors and other) to be used in organic synthesis. It should be noted that 3D printing with PE is hard to accomplish because of the low adhesion and considerable shrinkage of the material. All tested materials are resistant to water, acidic and basic aqueous solutions, and also to ethanol, which allows their exposure to aqueous reaction media. At the same time, PLA, PLA-Cu, ABS, SBS, Ceramo, HIPS and Primalloy parts are incompatible with acetone, ethyl acetate, toluene, DMF, THF and DCM.

Destruction scenario for a particular object is defined by the mode of interaction between the thermoplastics and the solvent at the solid-liquid interface, with predominance of either dissolution or swelling. Differential behavior of some materials depends on the solvent: for example, PLA dissolves in DCM, but delaminates in acetone.

Resistance of the printed parts to solvents can be increased by three ways. Firstly, the polymers can be modified by additives that would protect them from the solvent action. Secondly, the influence of solvents can be prevented by reasonable choice of 3D printing parameters, as decreased porosity prevents penetration of the solvent. Alternatively, the influence of solvents can be mitigated by adjustment of the part geometry.

## Methods

The materials used for the study included ABS, SBS, PLA, PLA-Cu, Nylon, Nylon-C, PP, PE, PETG, HIPS, POM, Ceramo, Primalloy, and photopolymer resin obtained from commercial sources.

### 3D printing

The parts were built by FDM with a Picaso 3D Designer Pro 250 printer at extrusion multiplier k = 0.9 (except for experiments with different k) and primary filament thickness of 1.75 mm. Printing with ABS, SBS, PLA, Nylon, PP, PE, PETG, HIPS, POM, and Primalloy was accomplished at a layer height of 0.2 mm by using a 0.3 mm nozzle. Printing with filled plastics PLA-Cu, Nylon-C, and Ceramo was accomplished by using a 0.5 mm nozzle at a layer height of 0.35 mm. The printing parameters (extruder temperature, build plate temperature, cooling intensity) are specified in Supplementary Table S1.

Printing with PE was carried out in a composite mode, with layers 1 to 10 printed at an extruder temperature of 250 °С without cooling. Starting from layer 11, the parameters were changed to 230 °С extruder temperature, 50% cooling, and 100 °С building plate temperature.

At a print speed of 40 mm/s with 100% infill density for all materials, the printing time constituted 12–14 minutes for a single part and <90 minutes for an array of 12 parts.

Setting of printing parameters and G-code were accomplished by using the Simplify3D 3.1.1 software (Simplify3D, LLC, 2016).

Three thermoplastics from the list, PLA-Cu, Nylon-C and Ceramo, contain microparticles as reinforcing components, whereas Primalloy has well-defined elastomer properties. The identical stability tests were applied to photopolymer resin (used for modeling by the LCD photopolymerization) in the same shape of a hollow cylinder with a bottom.

FDM with PLA, PLA-Cu, ABS, SBS, Ceramo, HIPS, PETG and Primalloy proceeded smoothly. These materials are highly suitable for producing parts of the given size by 3D printing. Nylon (Taulman) and Nylon-C are also good for FDM of small objects, although some shrinkage is observed for these materials after the part is finished. In the case of POM, the shrinkage is more pronounced, even with such small objects. Another shortcoming of POM, its low adhesion to the building plate, can be overcome by applying a specific adhesive coating (glue). The shrinkage of PP, although considerable for bigger objects^55^, was negligible, but its adhesion to the building platform was too low even with the adhesive coating. Due to this complication, FDM with PP was carried out on a 3 mm thick PP sheet firmly attached to the 3D printer build plate. PE turned out to be the most inconvenient FDM material. Making a high-quality product from PE by using a conventional desktop 3D printer is an extremely difficult task: in addition to the very low adhesion of PE to the plate, it is very hard to optimize the thermal conditions so that the part retains its shape during printing.

FDM copies of the conventional filament with 2.85 mm diameter were printed at different k values by using a 0.3 mm nozzle.

### Chemical resistance study

A 3D printed object was placed into an empty vessel of 30 mm outer diameter and 70 mm height. The object was topped with a steel bead (in the experiments with ethanol, ethyl acetate, acetone, toluene, dimethylformamide, tetrahydrofuran, triethylamine, and dichloromethane) or a glass bead (in the experiments with acetic acid, water and aqueous solutions of NaOH 1 M, and H_2_SO_4_ 0.5 М). Steel bead had weight 11.9 g and diameter 14.3 mm; glass bead had weight 5.7 ± 0.3 g and diameter 16.1 ± 0.4 mm. The vessel was gently filled with 20 mL of solvent and closed tightly with a lid. In the experiments on resistance of FDM parts made of different materials, the snapshots were made every 6 seconds in the course of 1 hour (Supplementary Figs. S1–S4, S9–S20, S22–43) Time-lapse photography was performed in automatic mode using a Nikon D610 camera with AF-S Micro NIKKOR 60 mm lens. Parameters of photography were the following: focal number f/4, ISO 400, and shutter speed 1/200 s.

After the experiments, the area of FDM parts in the images was analyzed at every 3 minutes using program algorithm of CAD software. The resistance of FDM parts was assessed by deviation of the area from the initial size.

In the experiments on the effect of Archimedean force, the snapshots were made every 6 seconds for 2.5 hours (Supplementary Fig. S45). The FDM parts were printed from PLA and immersed in DCM.

To study the effect of the indicator load shape, a ball of stainless steel and a brass cylinder were compared in experiments on resistance of PLA parts in DCM. The indicators had the same weights. The snapshots were made every 6 seconds in the course of 1.5 hours (Supplementary Fig. S46 and Movie S5).

In the experiments on resistance of FDM cylindrical parts printed at different values of extrusion multiplier, the snapshots were made every 6 seconds for 1.0 hours (Supplementary Figs. S49, S50). The FDM parts were printed from PLA and immersed in DCM.

In the experiments on resistance of individual FDM parts for detailed study of destruction patterns, the snapshots were made every 3 seconds in the course of 80 minutes (Figs. 2a and 4).

In the experiment on washing-out of the ingredients from FDM part printed from PLA-Cu in acetone, a special mount allowing the use of magnetic stirrer was applied (Supplementary Fig. S44).

In the experiments on the effect of extrusion multiplier k on structural stability of FDM parts, a series of PLA and ABS test tubes with 0.6 mm wall thickness was printed at different values of k (0.6; 0.7; 0.8; 0.9; 1.1) by using a 0.3 mm nozzle. To test the resistance, the printed test tubes were filled with ~3.7 ml of dichloromethane. A 36.6 g brass cylinder was used as indicator of the structural integrity loss. The duration of the experiment was 1 hour in each case. The snapshots were made every 6 seconds. In this work, thin-walled 3D-printed parts were analyzed because chemical labware includes thin walled items. That’s why influence of the infill density on the resistance of the FDM parts were not analyzed.

### Microscopy study

Morphology of collapsing FDM part surfaces (PLA-Cu in DCM, PLA-Cu in acetone, and SBS in DCM) was analyzed using FE-SEM. A target-oriented approach was utilized for optimization of the analytic measurements^56^. Before the measurements, the samples were mounted on a 25 mm aluminum specimen stub and fixed with a double-sided carbon adhesive tape. Metal coating with a 7 nm film of 80/20 platinum/palladium alloy by using magnetron sputtering was performed as described earlier^57^. The observations were carried out using a Hitachi SU8000 field-emission scanning electron microscope (FE-SEM). The images were acquired in a secondary electron mode at 2 kV accelerating voltage and 8–10 mm working distance. Morphology of the samples was studied taking into account the possible influence of metal coating. Study of morphology of Primalloy part swelling in toluene was conducted using stereo microscope Leica M125 (Supplementary Fig. S21).

## Supplementary information

Supplementary Movie 1

Supplementary Movie 2

Supplementary Movie 3

Supplementary Movie 4

Supplementary Movie 5

Supplementary information

## Data Availability

All data generated or analyzed during this study are included in this published article (and its Supplementary Information files).
