# Supplementary material for: Revealing interactions of layered polymeric materials at solid-liquid interface for building solvent compatibility charts for 3D printing applications
Source: Sci Rep. 2019 Dec 27;9:20177. doi: 10.1038/s41598-019-56350-w (PMC6934857; doi:10.1038/s41598-019-56350-w)
Supplement: Supplementary file 6 — Supplementary information [file 41598_2019_56350_MOESM6_ESM.pdf]

Supporting Information

for the article

Revealing interactions of layered polymeric materials at solid-liquid interface for building solvent compatibility charts for 3D printing applications

Kirill S. Erokhin, Evgeniy G. Gordeev, Valentine P. Ananikov*

Zelinsky Institute of Organic Chemistry, Russian Academy of Sciences, Leninsky prospekt 47, Moscow, 119991, Russia; *val@ioc.ac.ru*

Content

1. Description of videos 2
2. Parameters of 3d printing of the test parts 3
3. Experiments in dichloromethane media 4
4. Experiments in tetrahydrofuran media 8
5. Experiments in acetone media 10
6. Experiments in dimethylformamide media 12
7. Experiments in toluene media 14
8. Experiments in ethyl acetate media 16
9. Experiments in triethylamine media 18
10. Experiments in acetic acid media 20
11. Experiments in ethanol media 22
12. Experiments in media of water solution of acid (H_2SO_4 0.5M) 24
13. Experiments in media of water solution of base (NaOH 1M) 26
14. Experiments in water media 28
15. Total table of FDM materials stability 30
16. Experiment on ingredient outwashing from PLA-Cu in acetone media 31
17. Study of effect of Archimede’s buoyant force 32
18. Study of influence of indicator shape 33
19. Influence of the extrusion multiplier on the structural stability of FDM parts in dichloromethane media 34
20. Comparison of resistance of the extruded part and FDM parts 36

1. Description of videos

Supplementary_Movie_S1.mp4

This video illustrates the effect of the extrusion multiplier on the stability of FDM parts made of PLA in dichloromethane. For comparison, the effect of the solvent on the extruded part is shown. All parts have the same dimensions and similar weight. Three FDM parts were manufactured with different extrusion multiplier values: 0.8; 0.9; 1.0. That is, this experiment demonstrates the stability of FDM parts manufactured with extrusion multipliers that are most commonly used in practice of FDM printing. A brass cylinder was used as an indicator. A video is a time-lapse shot taken at 6 seconds intervals during 1.0 hour. During the shooting, 600 individual frames were obtained, which were then combined into this video at a frame rate of 25 fps.

Supplementary_Movie_S2.mp4

This video also demonstrates the effect of the extrusion multiplier on the stability of FDM parts made of PLA in DCM media. Unlike the Movie-S1 video, this video shows parts made with increased extrusion multipliers: 1.1; 1.2; 1.3. An extruded part is selected for comparison. All parts are characterized by almost the same dimensions and weights. The indicator is a brass cylinder. A video is a time-lapse shot taken at 6 seconds intervals during 1.0 hours. During the shooting, 600 individual frames were obtained, which were then combined into this video at a 25 fps frame rate.

Supplementary_Movie_S3.mp4

This video shows various types of degradation of FDM products made from various materials. *Disintegration* of the FDM parts is shown by the example of a PLA material filled with copper particles in a methylene chloride medium. *Delamination* is displayed using PLA filled with copper particles in acetone. *True dissolution* is characteristic of SBS in methylene chloride, and *swelling* is shown by the example of Primalloy material in toluene. All destruction models presented in this video were shot using the time-lapse method of photoshooting. The time-lapse was 3 seconds; the total duration of the shooting was 80 minutes. The resulting 1600 frames were combined into a video with a 25 fps frame rate.

Supplementary_Movie_S4.mp4

This video shows the absence of the influence of Archimedes force on the destruction time of FDM parts when using indicator beads of different masses. In the experiment steel beads and glass beads of the same size and FDM parts made of PLA were used. The experiment was performed in methylene chloride media. Three different volumes of solvent were used for each type of beads: a small volume into which a smaller part of the bead was immersed; the average volume into which most of the bead was immersed; the large volume into which the entire bead was immersed. In all cases, the destruction of the part occurs at about the same time. The video was obtained as a result of time-lapse shooting at 6 seconds intervals during 2.5 hours. Obtained 1500 frames were combined in a video with a 25 fps frame rate.

Supplementary_Movie_S5.mp4

This video shows the absence of the influence of the shape of the indicator loading on the dynamics of the destruction of FDM parts. FDM parts are made of PLA. The tests were carried out in methylene chloride media. A steel bead and a brass cylinder were used as an indicator loading. Both loads have the same weight. The loss of part integrity in experiments with different loads occurs almost simultaneously. The video is the result of time-lapse shooting, taken at 6 seconds time-lapse during 1.5 hours. The resulting 900 frames were combined in a video of 25 fps frame rate.

2. Parameters of 3d printing of the test parts

Table S1. FDM parameters used in this study for a set of materials.

Material	$T_{ext.}$, °C	T_{bed}, °C	Cooling, %
**ABS**	230	100	0
**SBS**	230	80	20
**PLA**	220	60	40
**PLA-Cu**	220	60	40
**Nylon**	245	100	0
**Nylon-C**	245	100	0
**PP**	235	80	20
**PETG**	240	80	20
**HIPS**	230	50	20
**POM**	190	100	0
**Ceramo**	255	100	20
**Primalloy**	235	80	0

3. Experiments in dichloromethane media

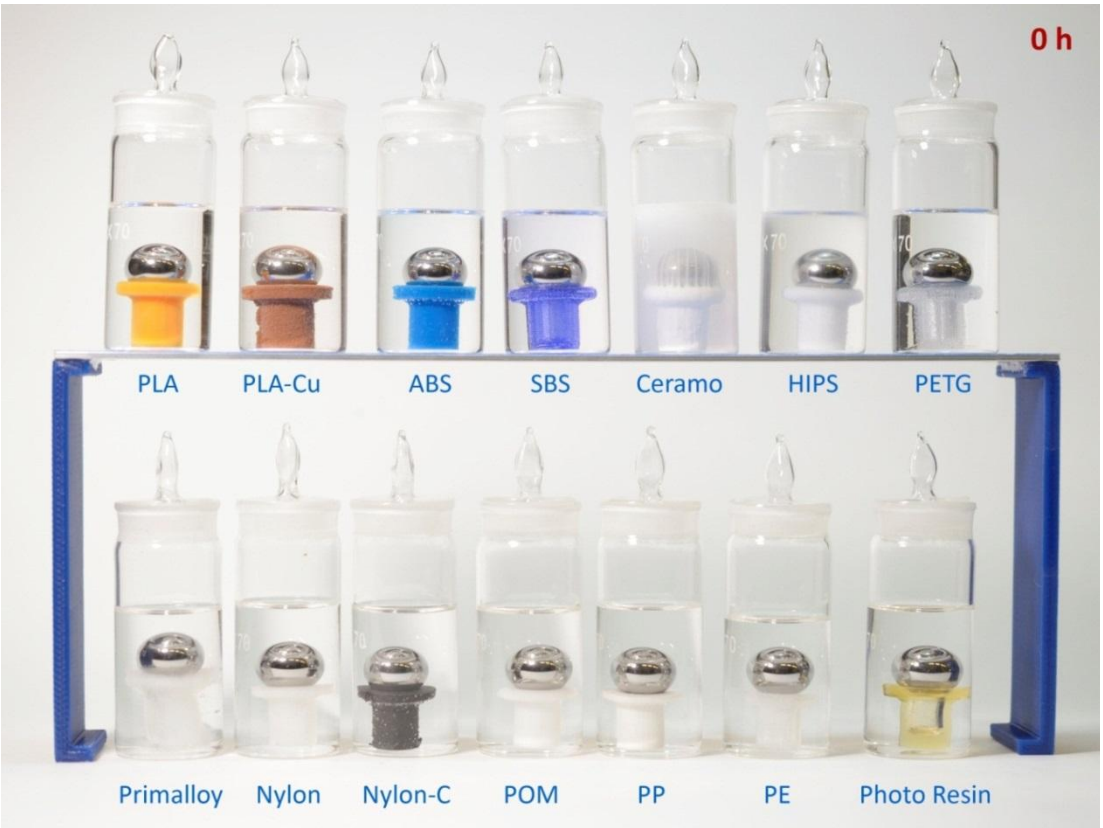

Figure S1. Snapshot of the experiment in DCM in the beginning.

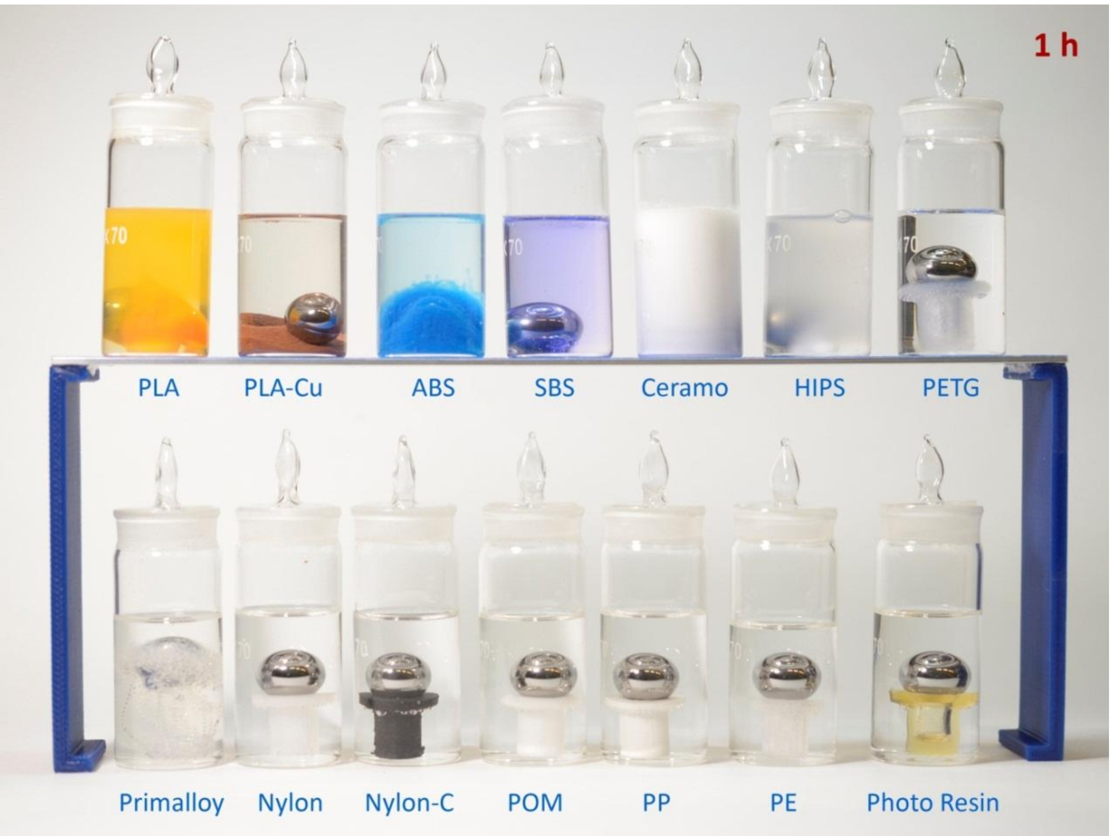

Figure S2. Snapshot of the experiment in DCM after 1h.

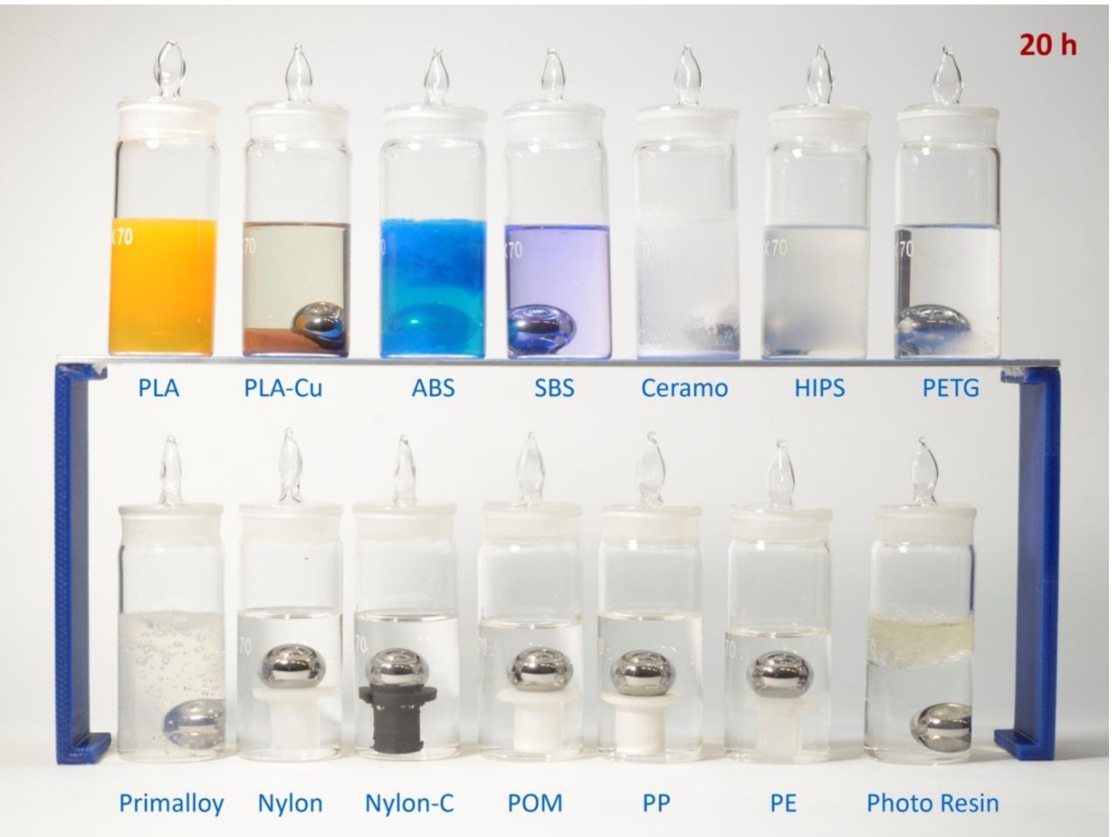

Figure S3. Snapshot of the experiment in DCM after 20 h.

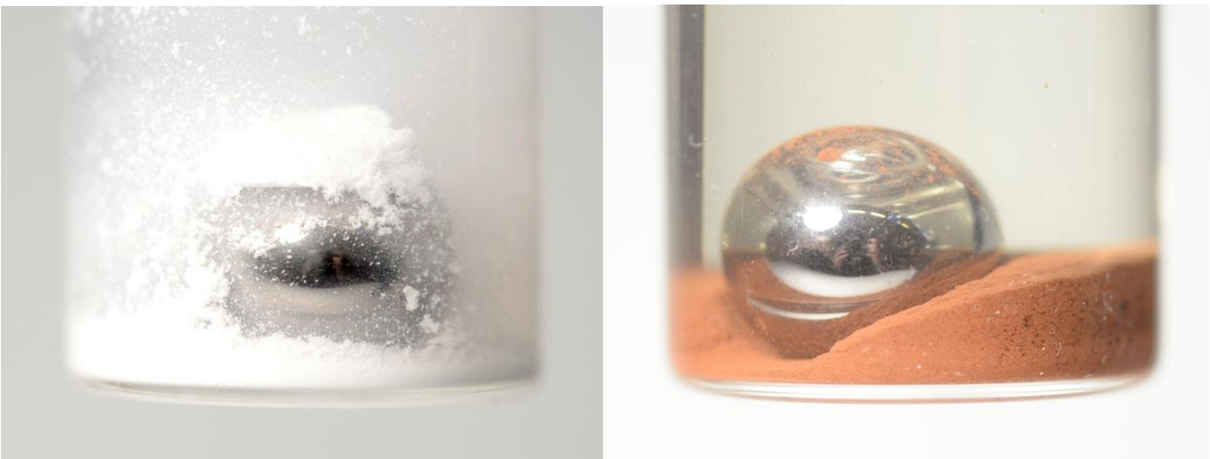

Figure S4. Snapshots of Ceramo and PLA-Cu parts in DCM after 20 h.

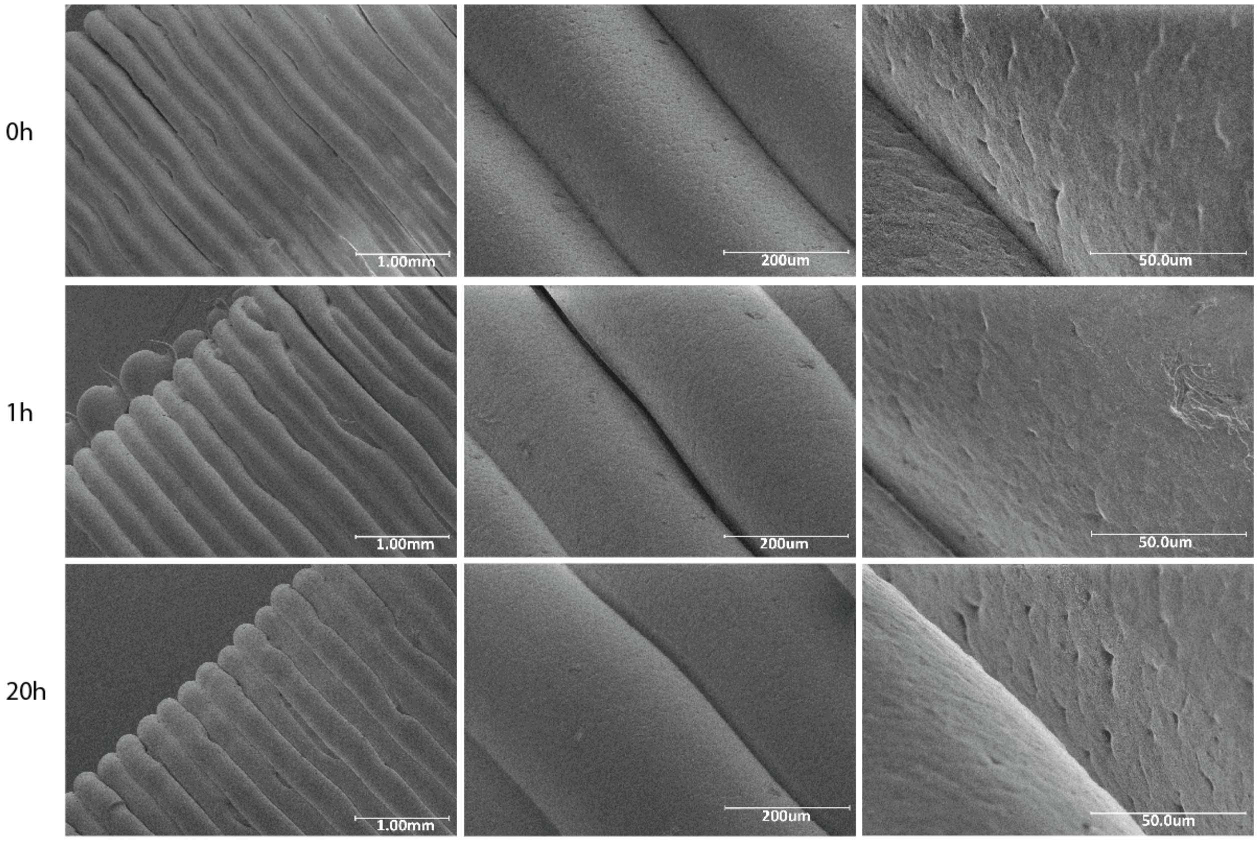

Figure S5. Microphotographs of surface of POM parts before experiment with DCM, after 1h and 20 h.

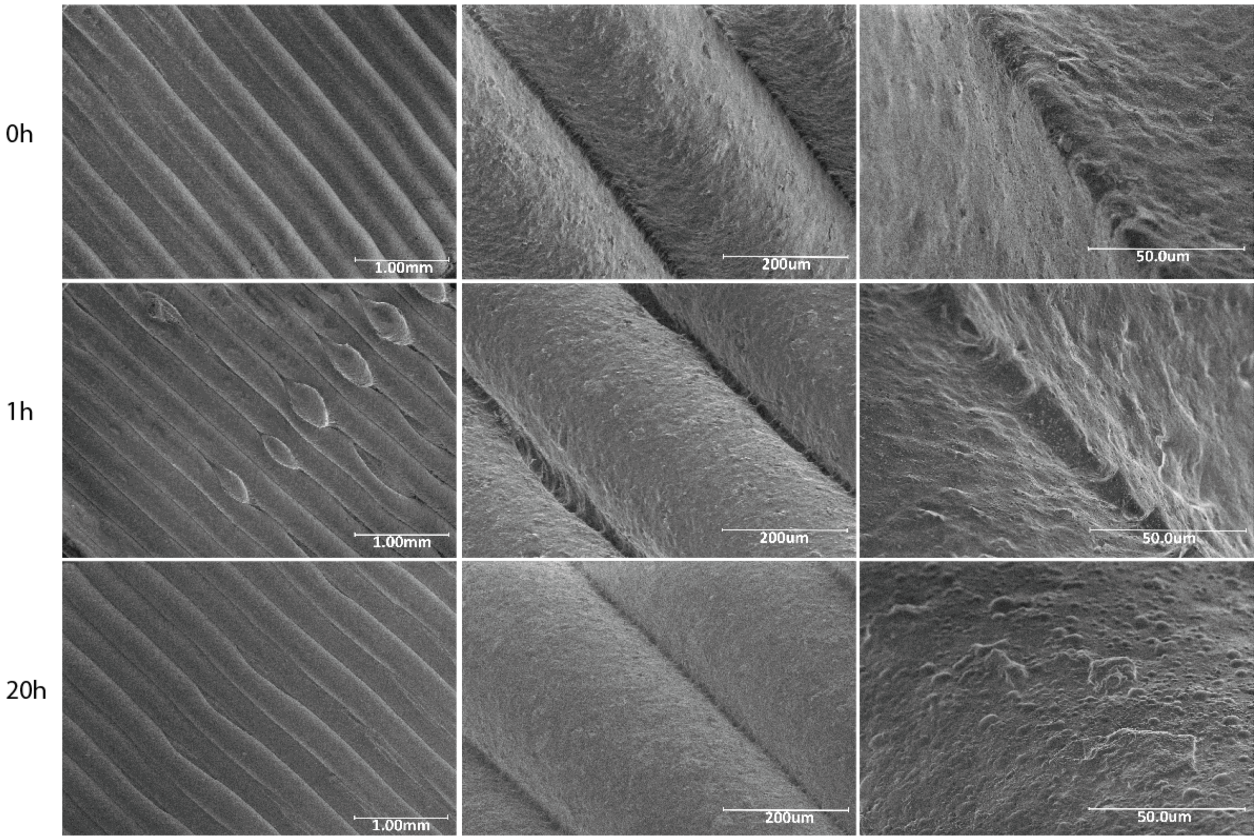

Figure S6. Microphotographs of surface of PP parts before experiment with DCM, after 1h and 20 h.

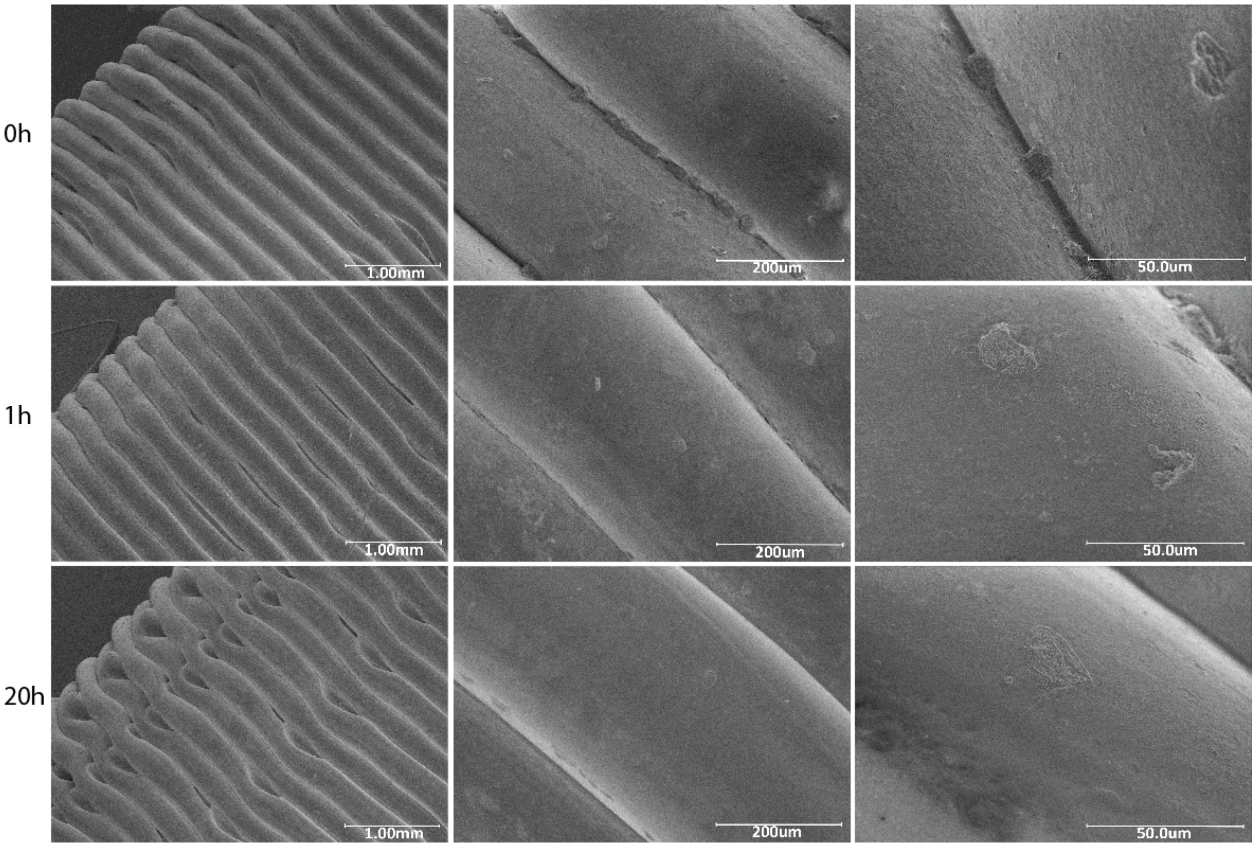

Figure S7. Microphotographs of surface of Nylon parts before experiment with DCM, after 1h and 20 h.

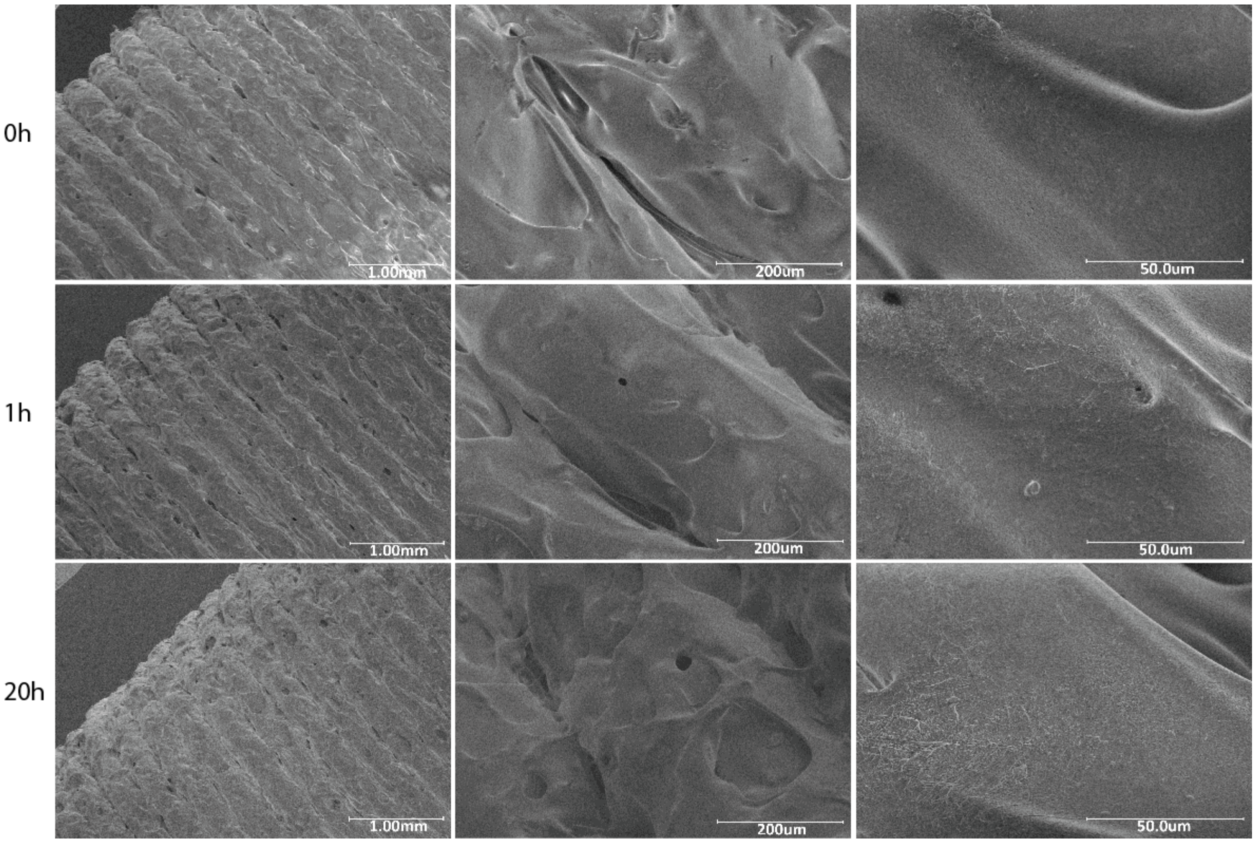

Figure S8. Microphotographs of surface of Nylon-C parts before experiment with DCM, after 1h and 20 h.

4. Experiments in tetrahydrofuran media

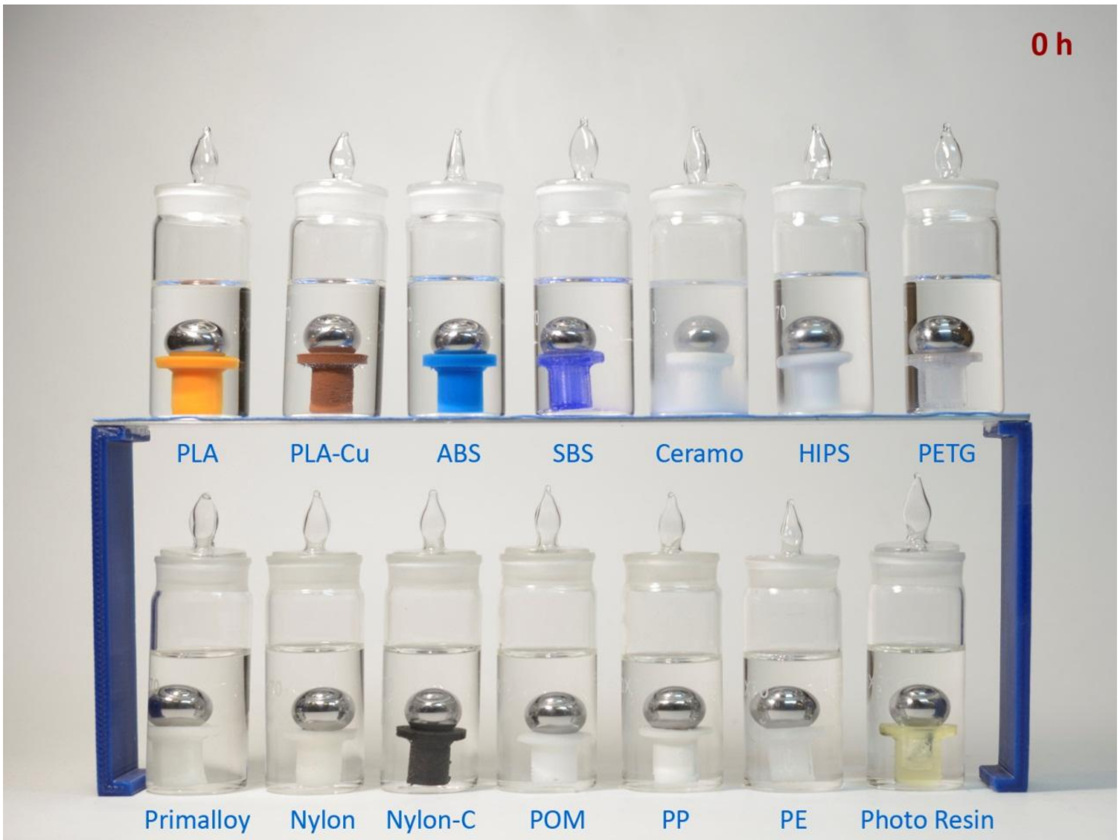

Figure S9. Snapshot of the experiment in THF in the beginning.

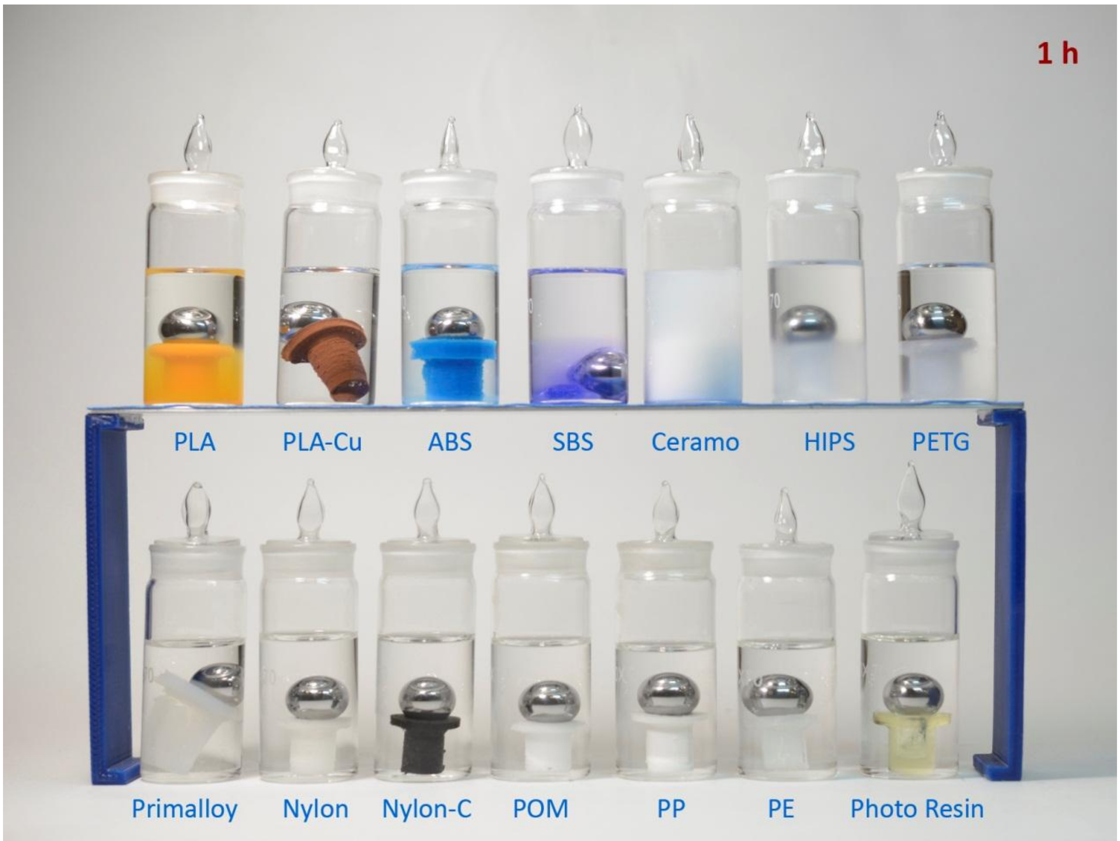

Figure S10. Snapshot of the experiment in THF after 1 h.

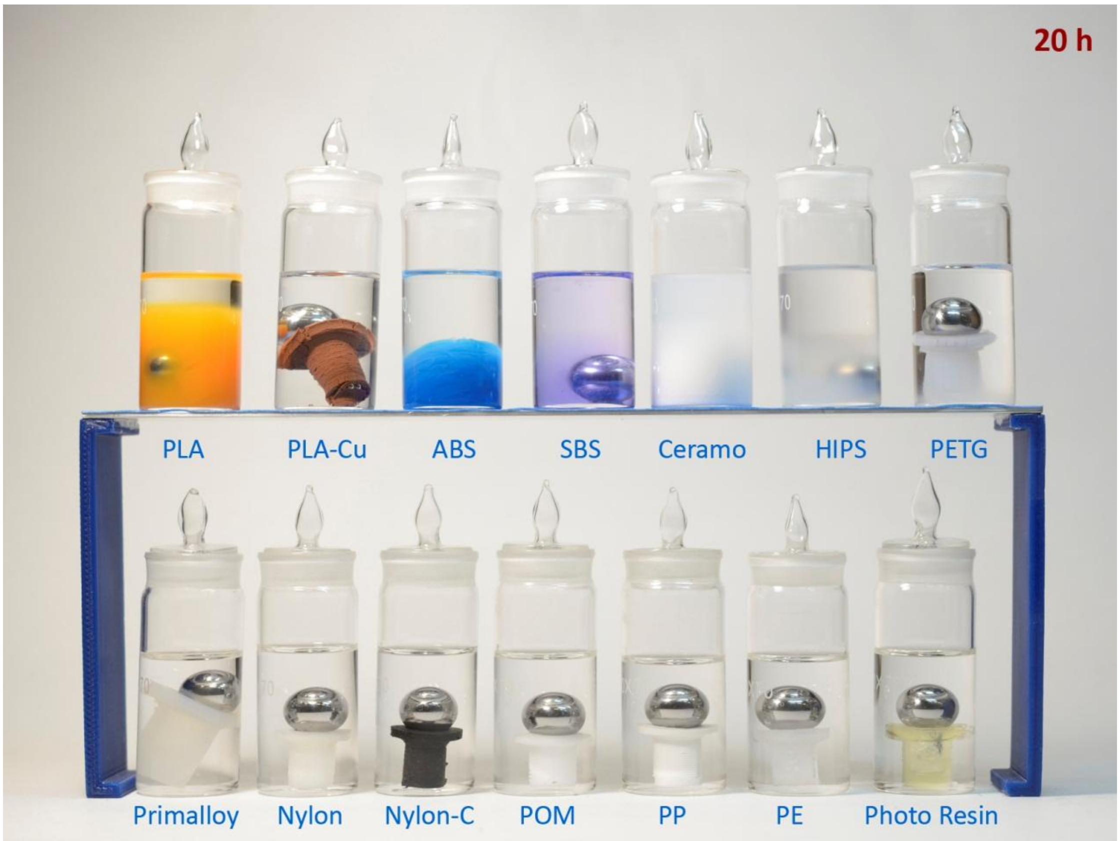

Figure S11. Snapshot of the experiment in THF after 20 h.

5. Experiments in acetone media

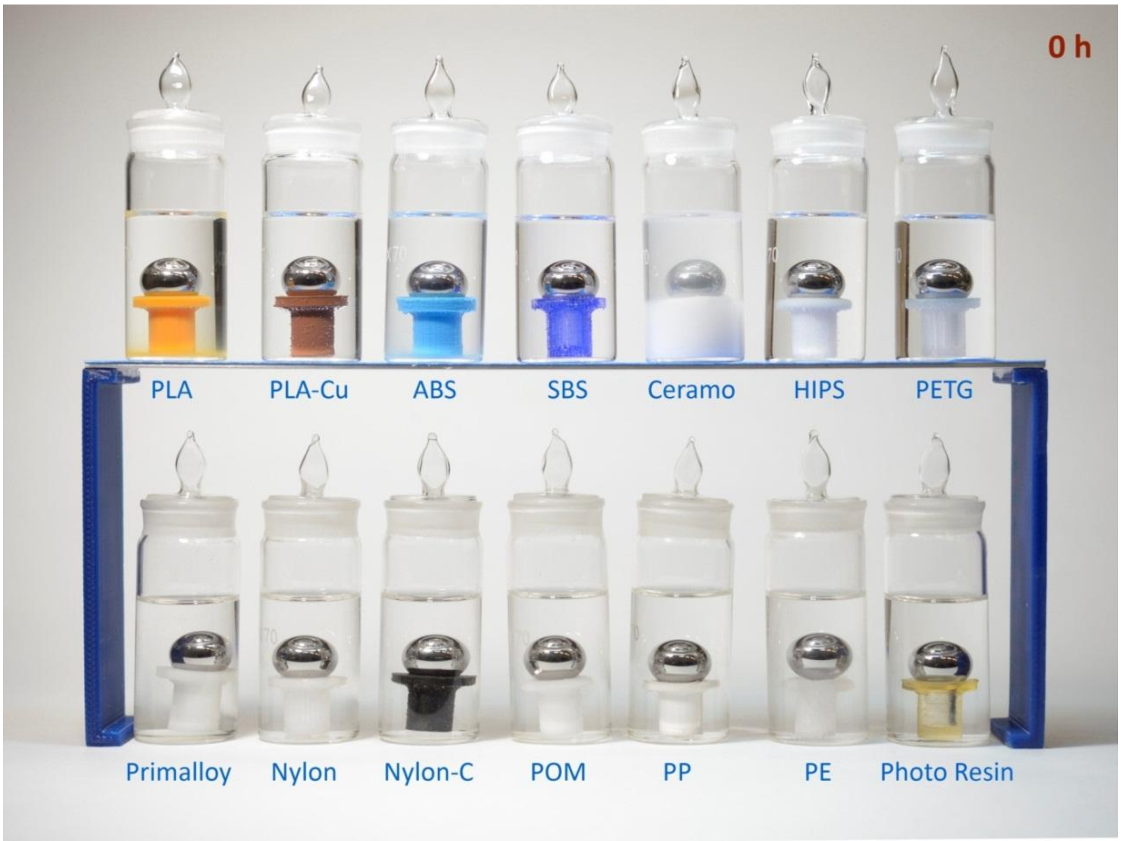

Figure S12. Snapshot of the experiment in acetone in the beginning.

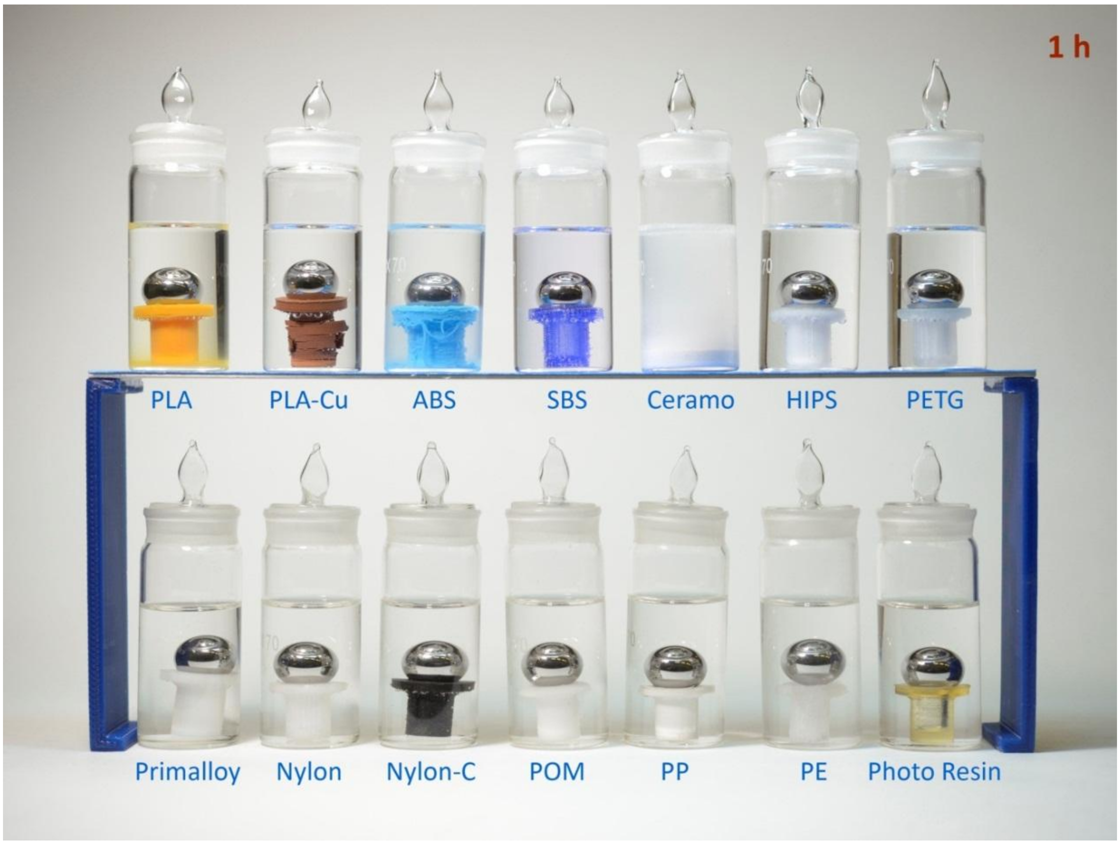

Figure S13. Snapshot of the experiment in acetone after 1 h.

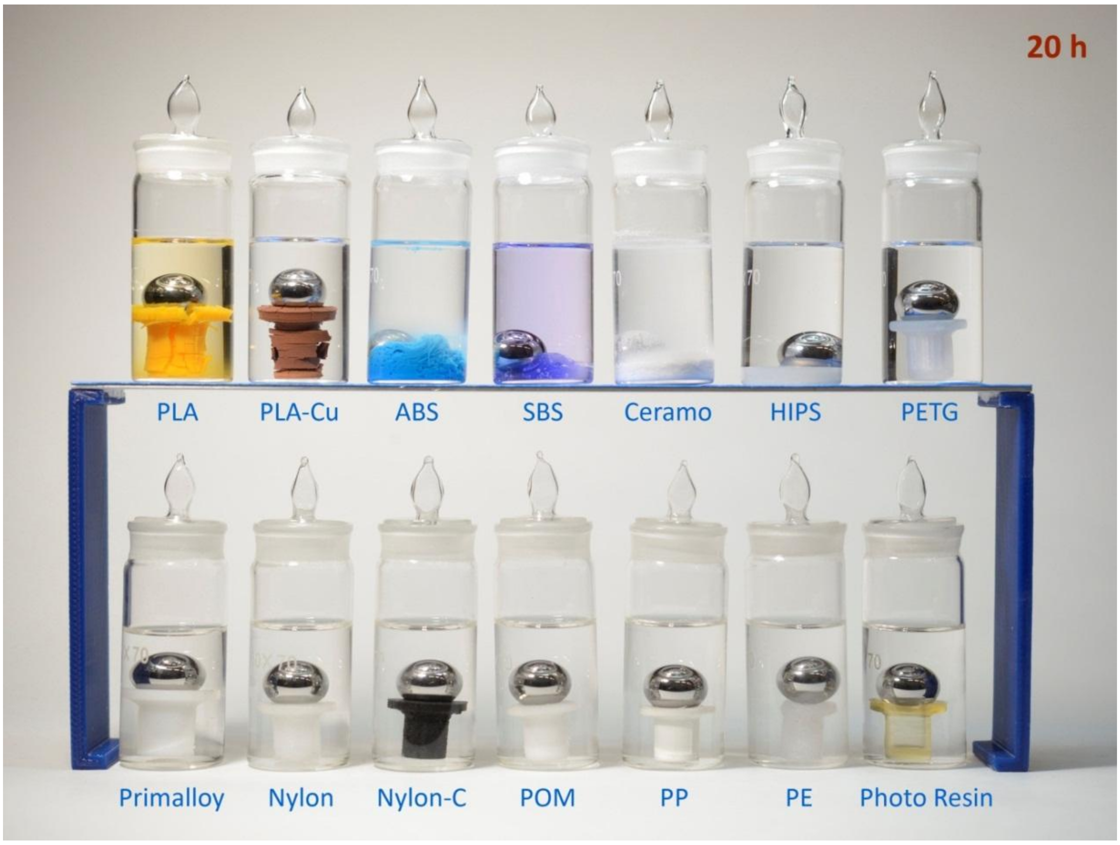

Figure S14. Snapshot of the experiment in acetone after 20 h.

6. Experiments in dimethylformamide media

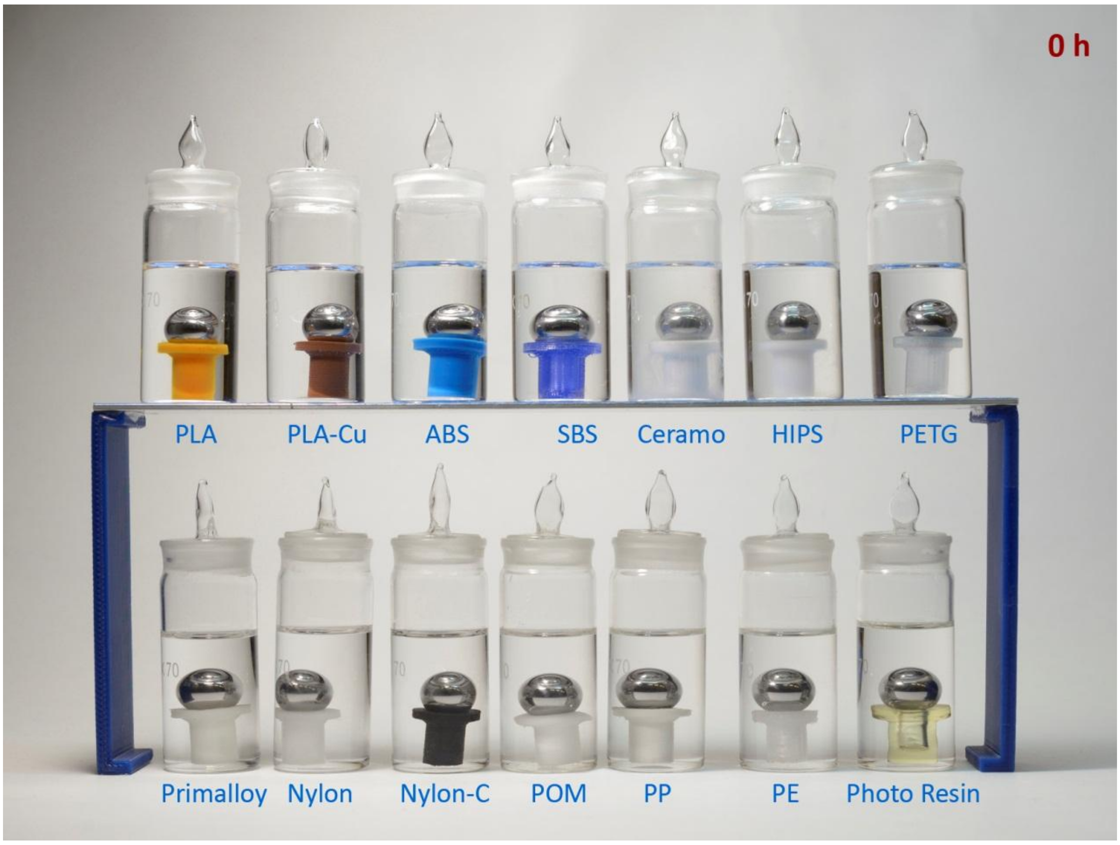

Figure S15. Snapshot of the experiment in DMF in the beginning.

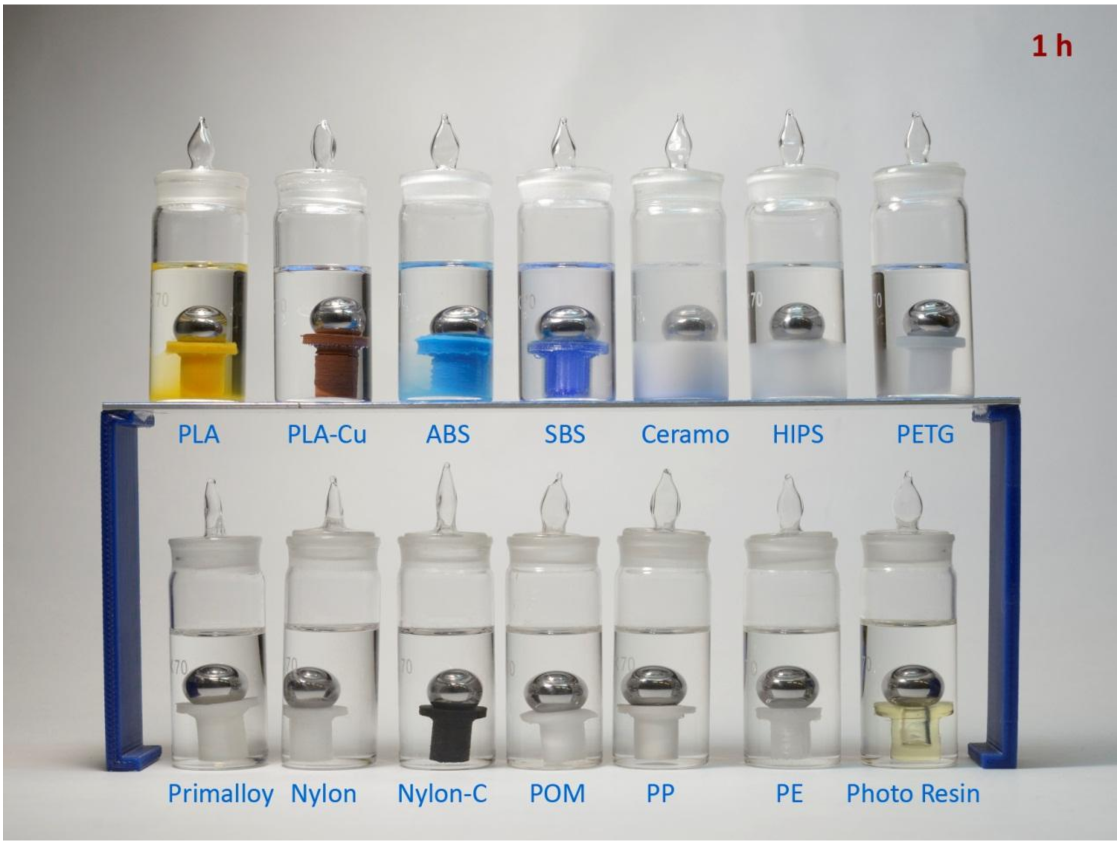

Figure S16. Snapshot of the experiment in DMF after 1 h.

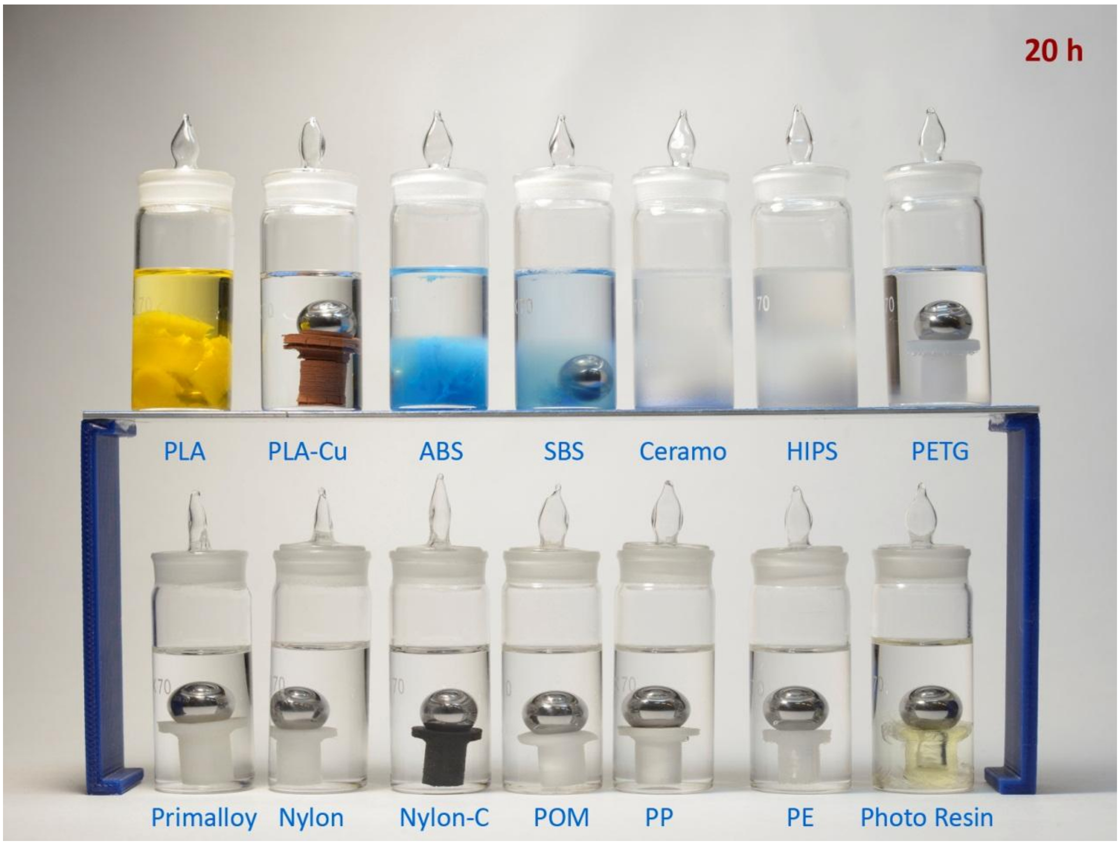

Figure S17. Snapshot of the experiment in DMF after 20 h.

7. Experiments in toluene media

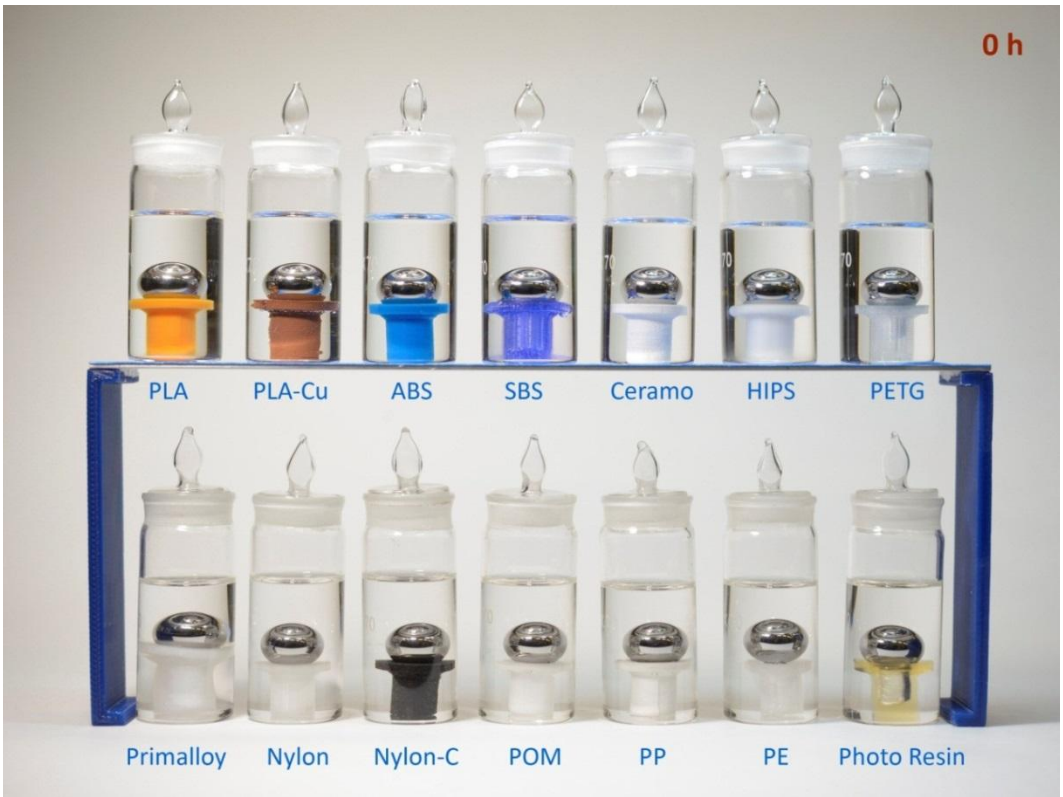

Figure S18. Snapshot of the experiment in toluene in the beginning.

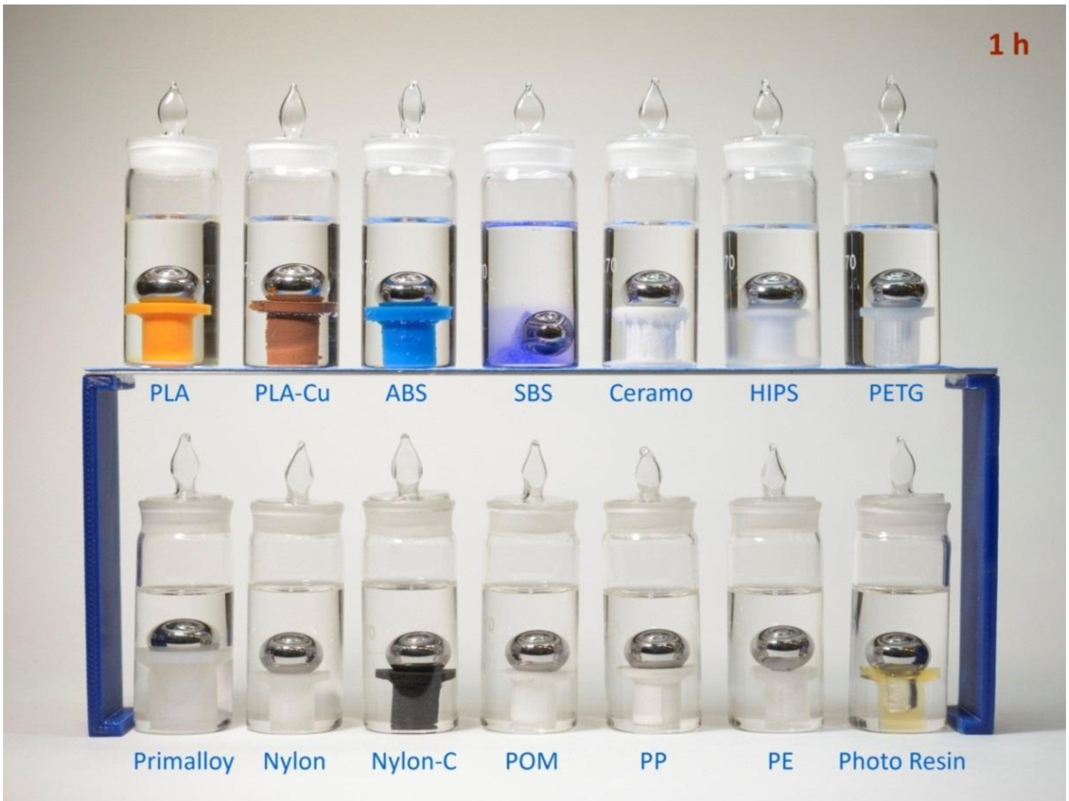

Figure S19. Snapshot of the experiment in toluene after 1 h.

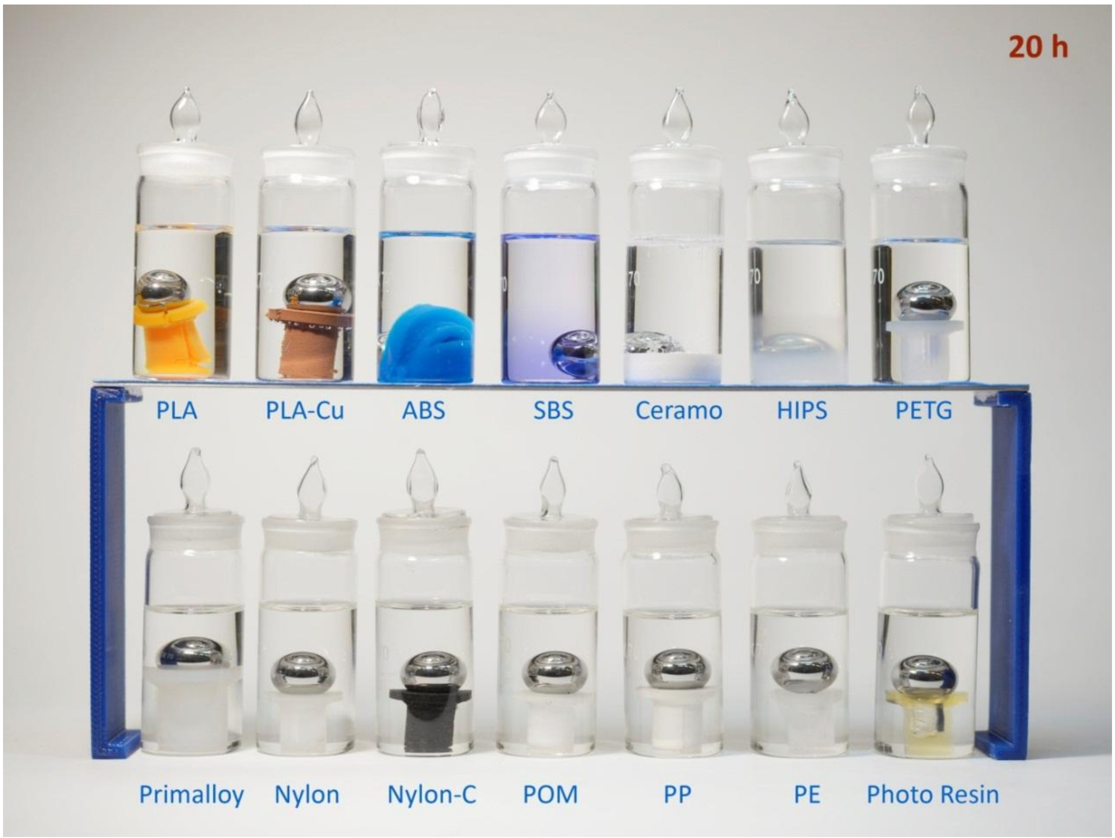

Figure S20. Snapshot of the experiment in toluene after 20 h.

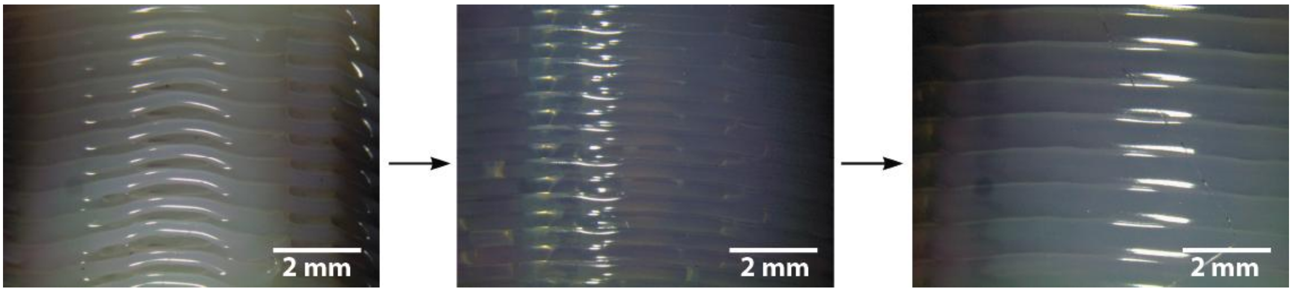

Figure S21. Microphotographs of surface of Primalloy parts during experiment with toluene.

8. Experiments in ethyl acetate media

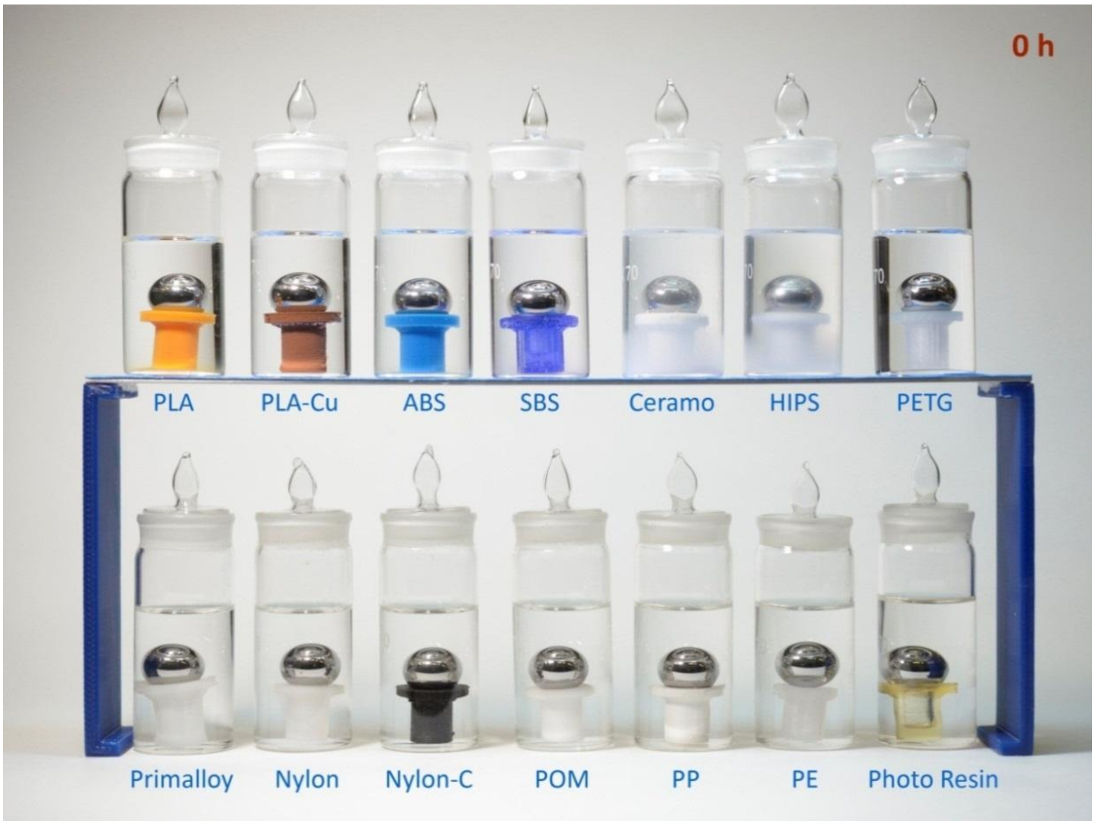

Figure S22. Snapshot of the experiment in ethyl acetate in the beginning.

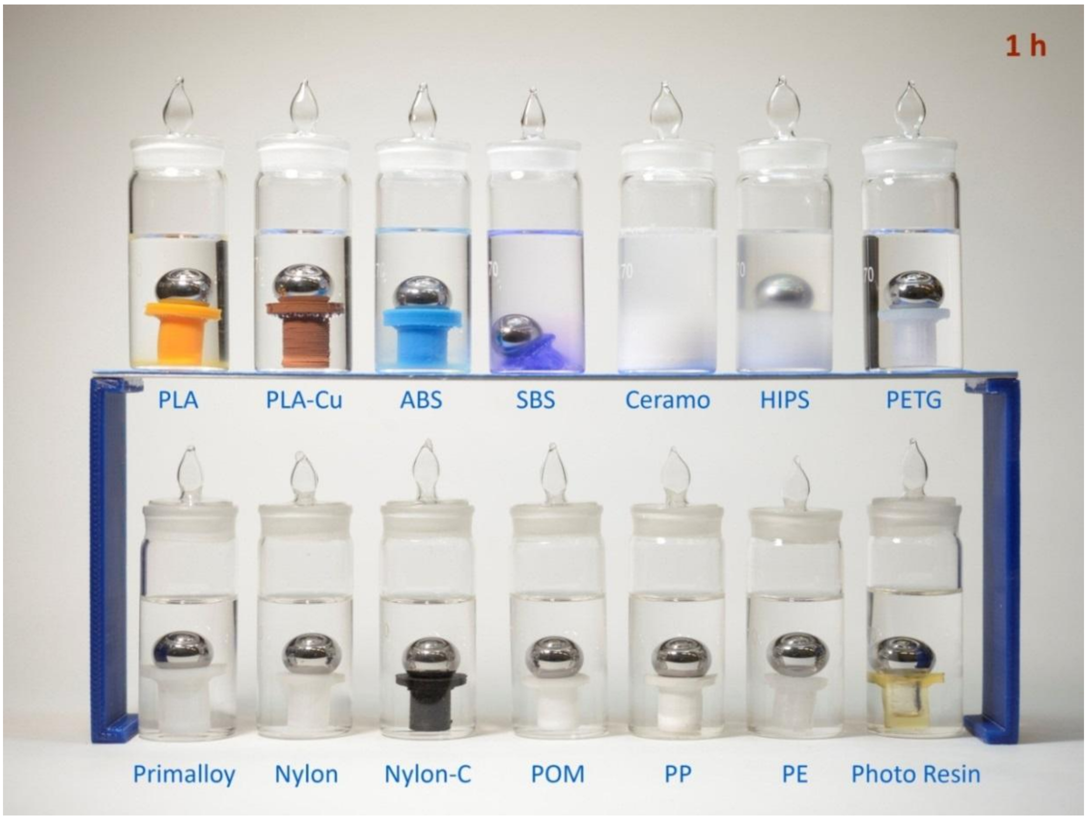

Figure S23. Snapshot of the experiment in ethyl acetate after 1 h.

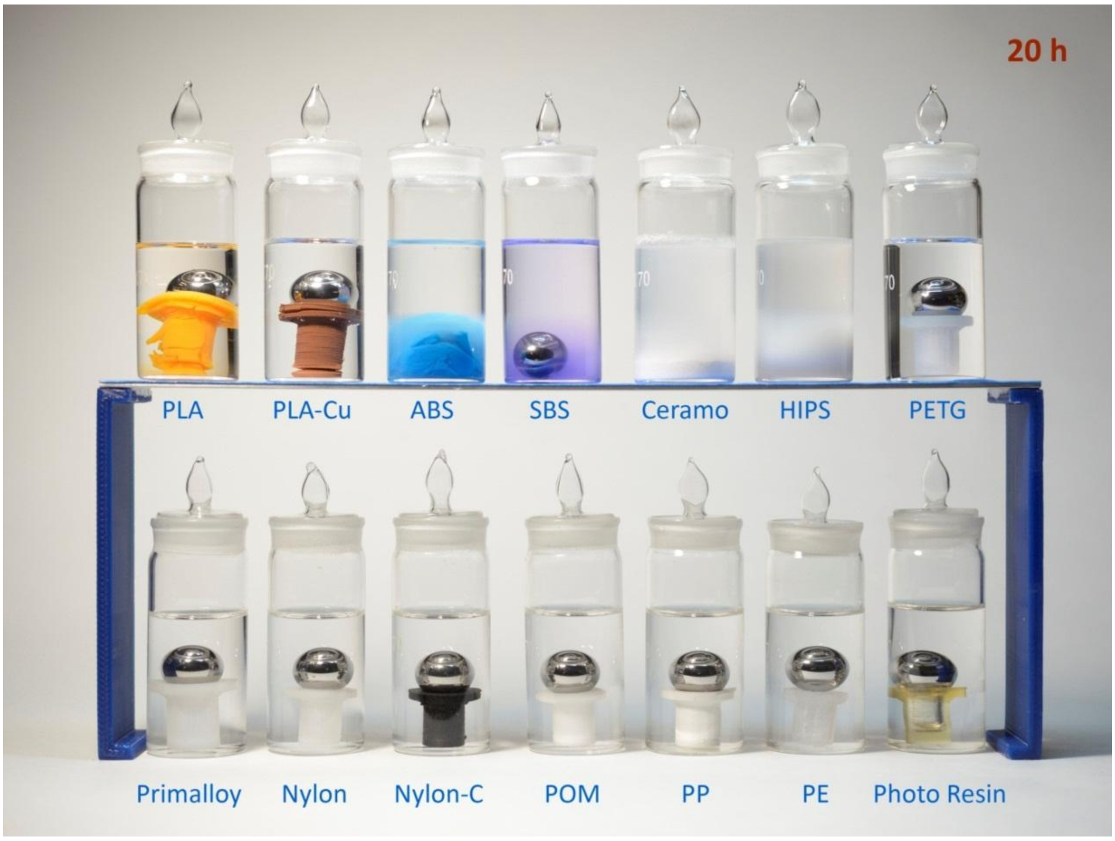

Figure S24. Snapshot of the experiment in ethyl acetate after 20 h.

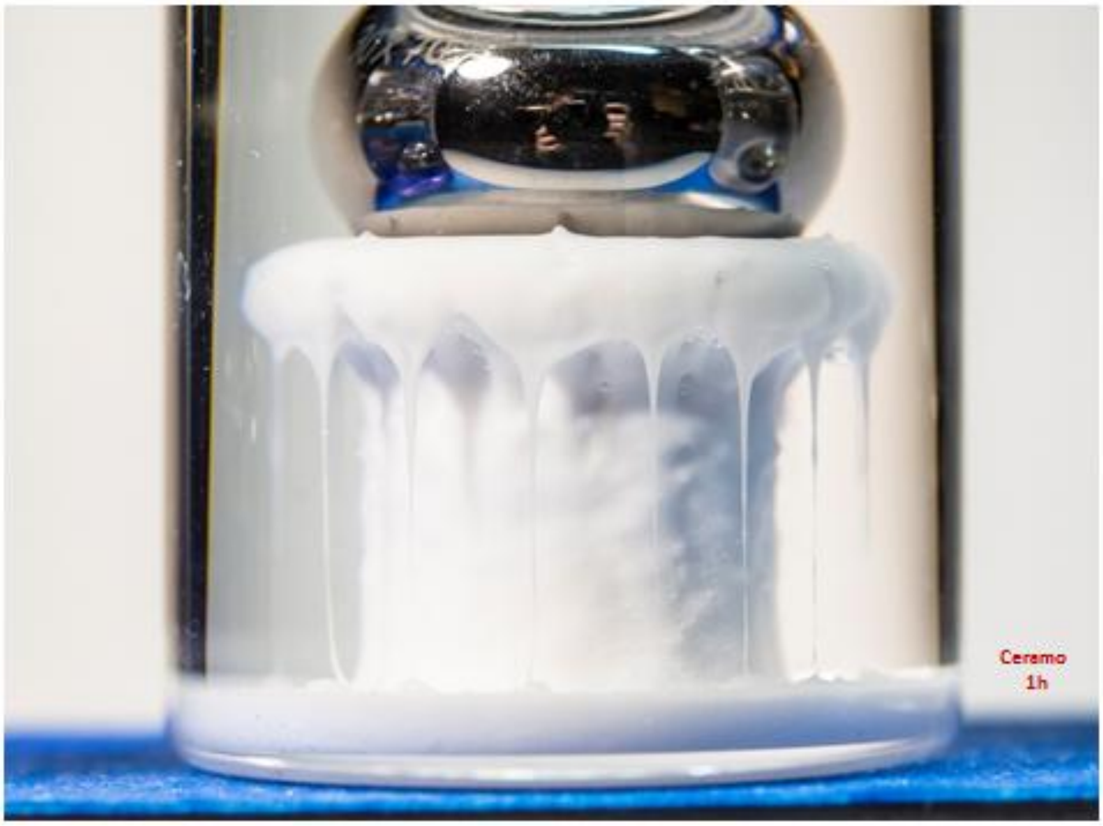

Figure S25. Snapshot of Ceramo part during the experiment.

9. Experiments in triethylamine media

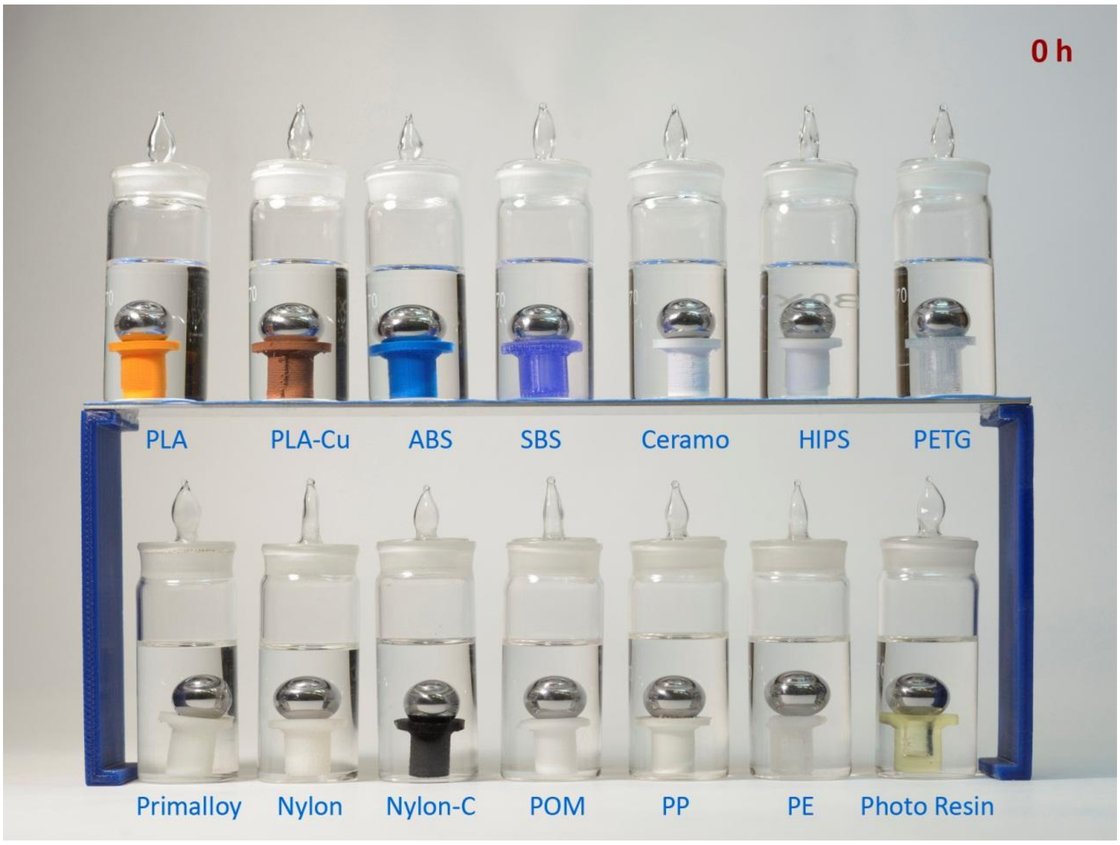

Figure S26. Snapshot of the experiment in triethylamine in the beginning.

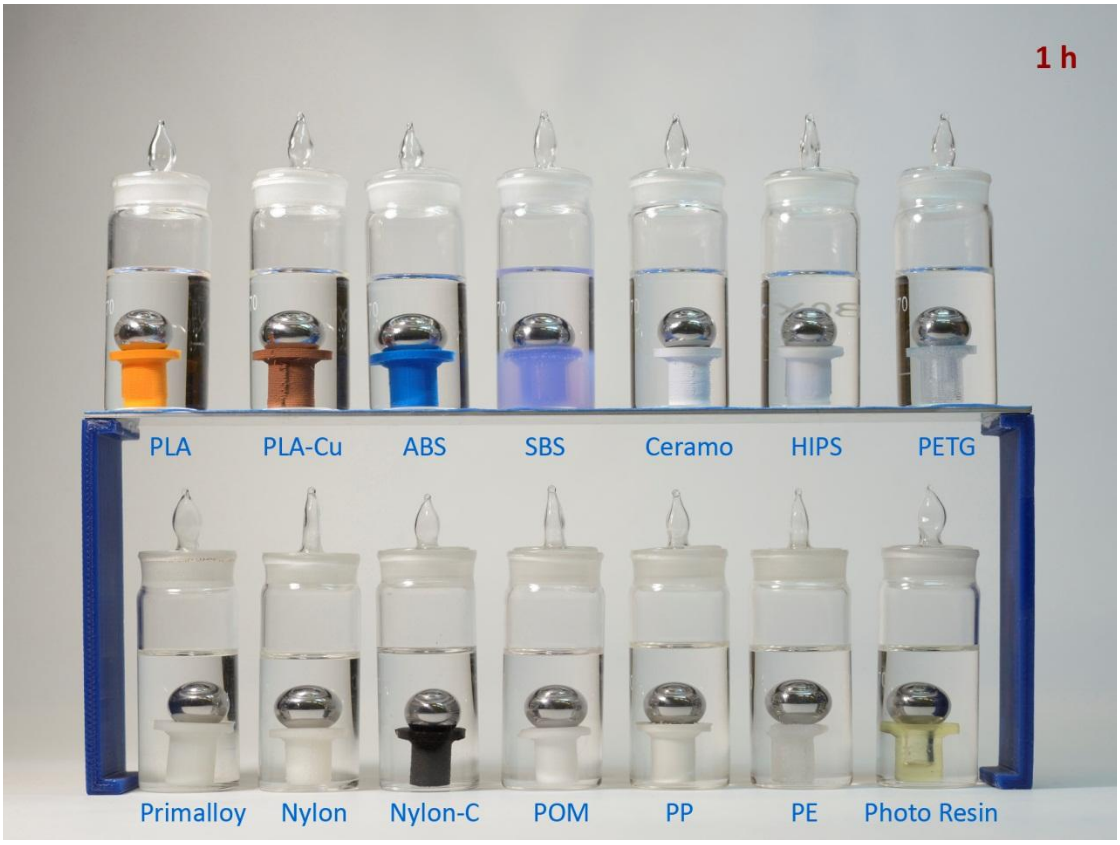

Figure S27. Snapshot of the experiment in triethylamine after 1 h.

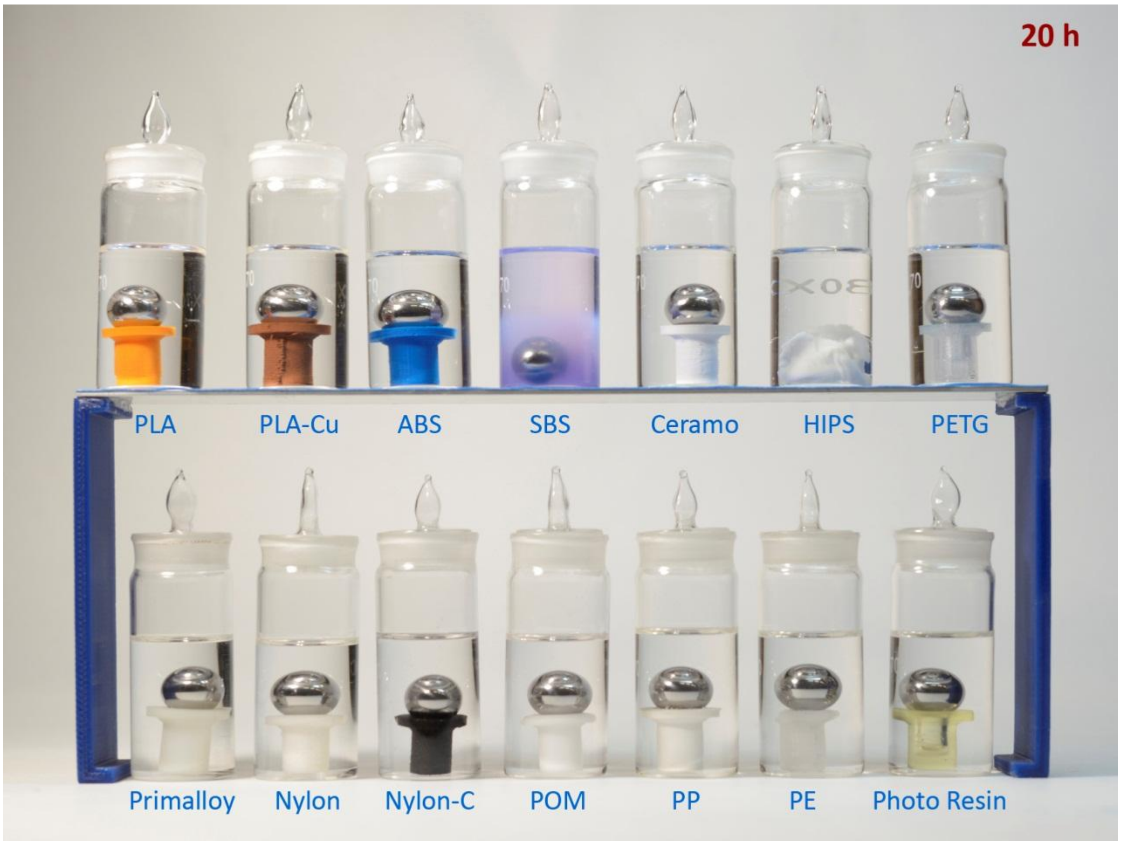

Figure S28. Snapshot of the experiment in triethylamine after 20 h.

10. Experiments in acetic acid media

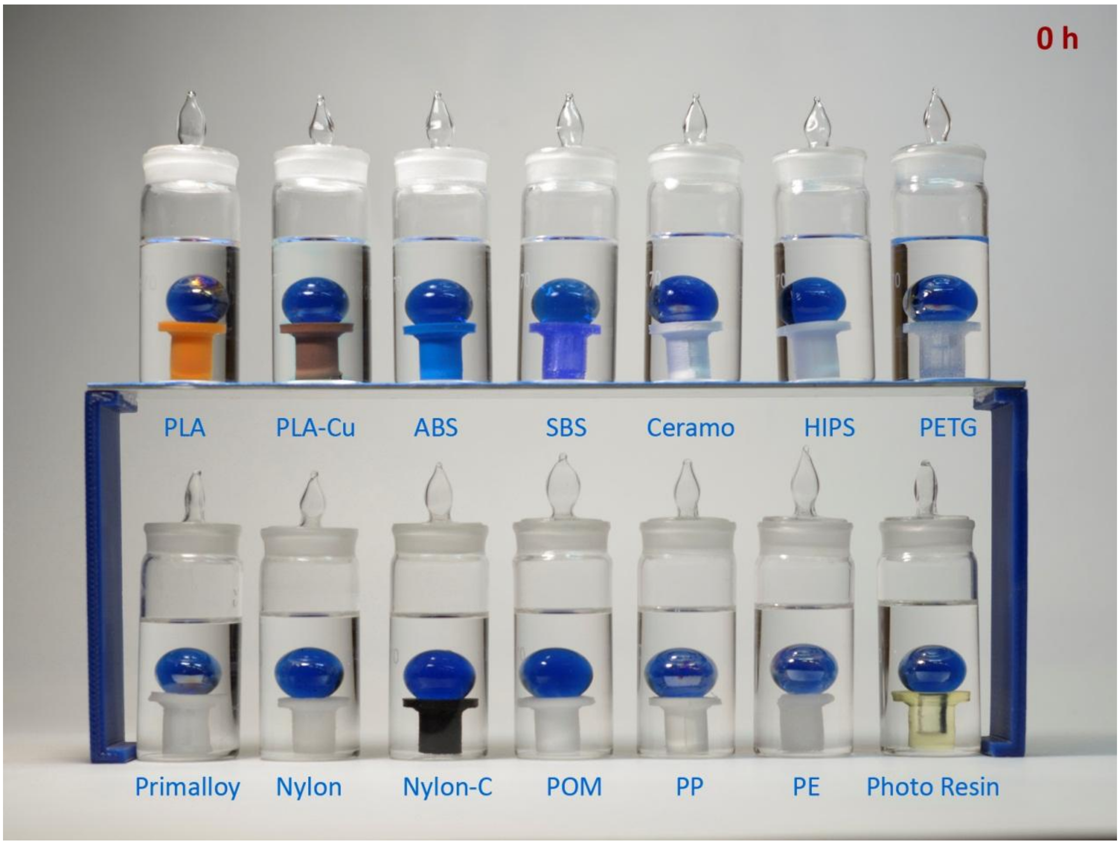

Figure S29. Snapshot of the experiment in acetic acid in the beginning.

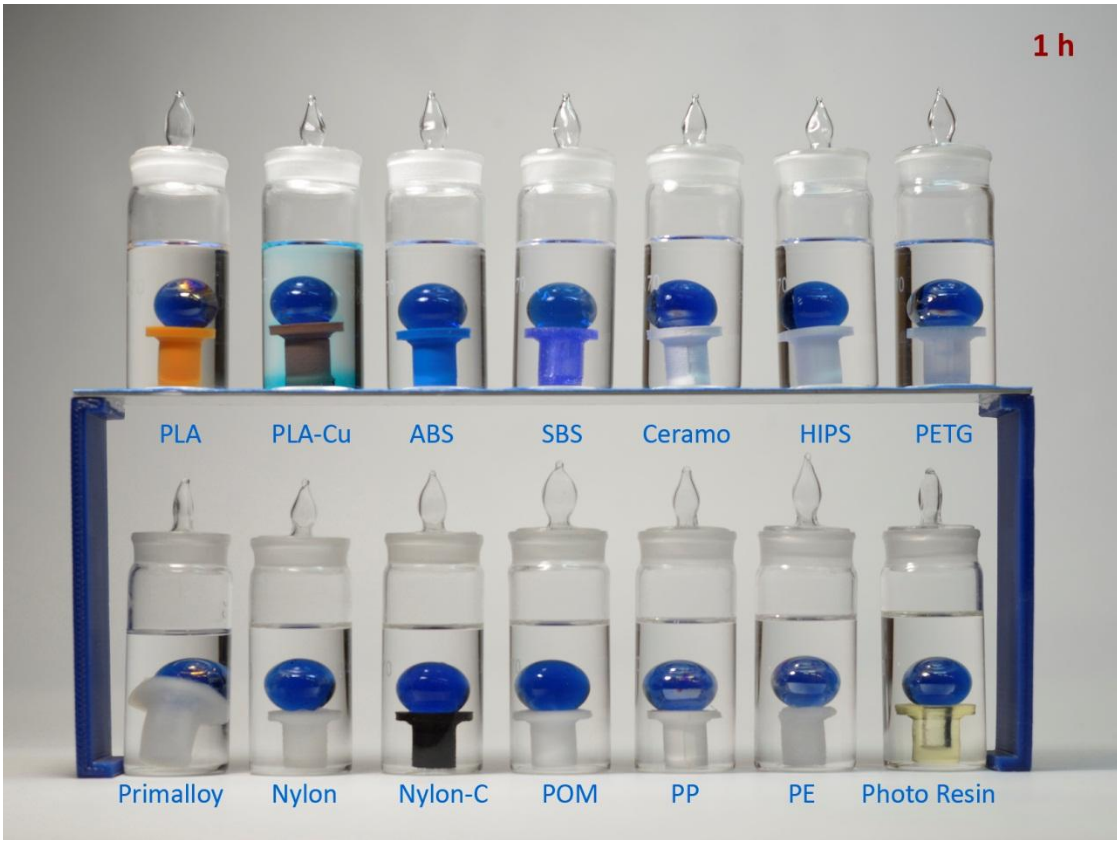

Figure S30. Snapshot of the experiment in acetic acid after 1 h.

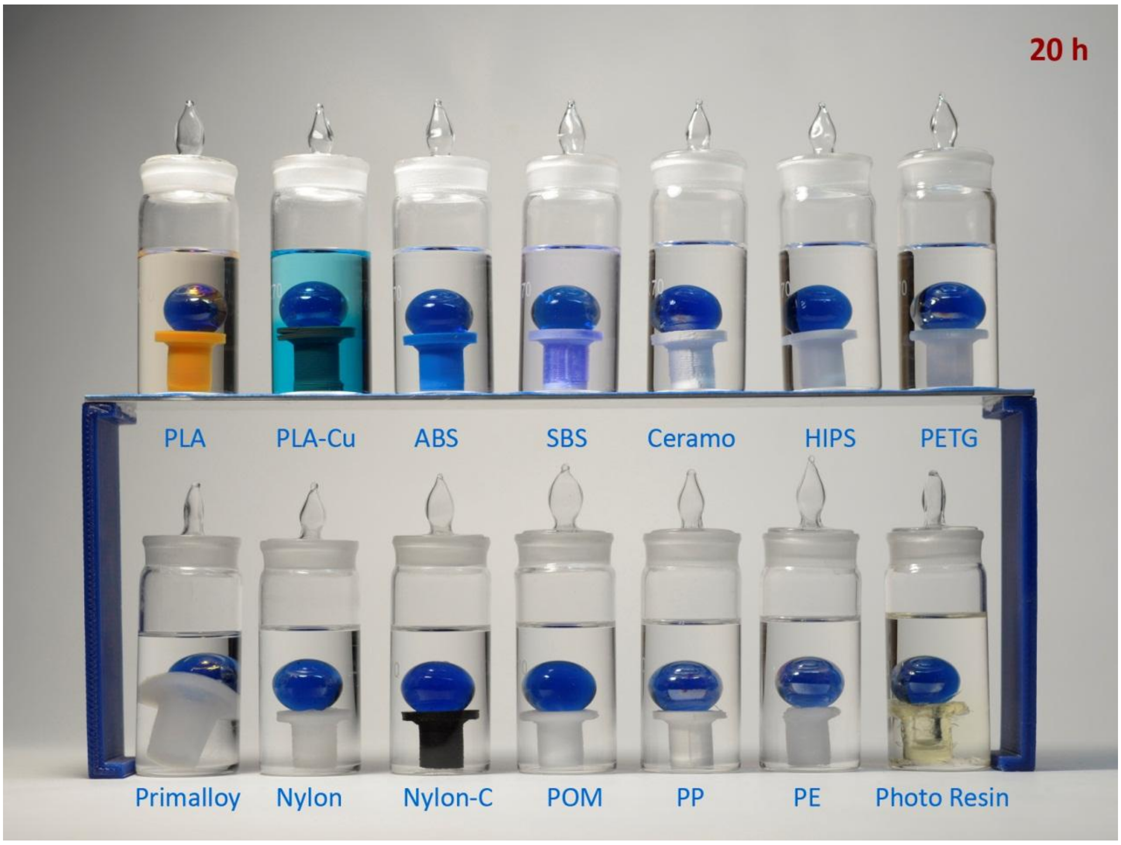

Figure S31. Snapshot of the experiment in acetic acid after 20 h.

11. Experiments in ethanol media

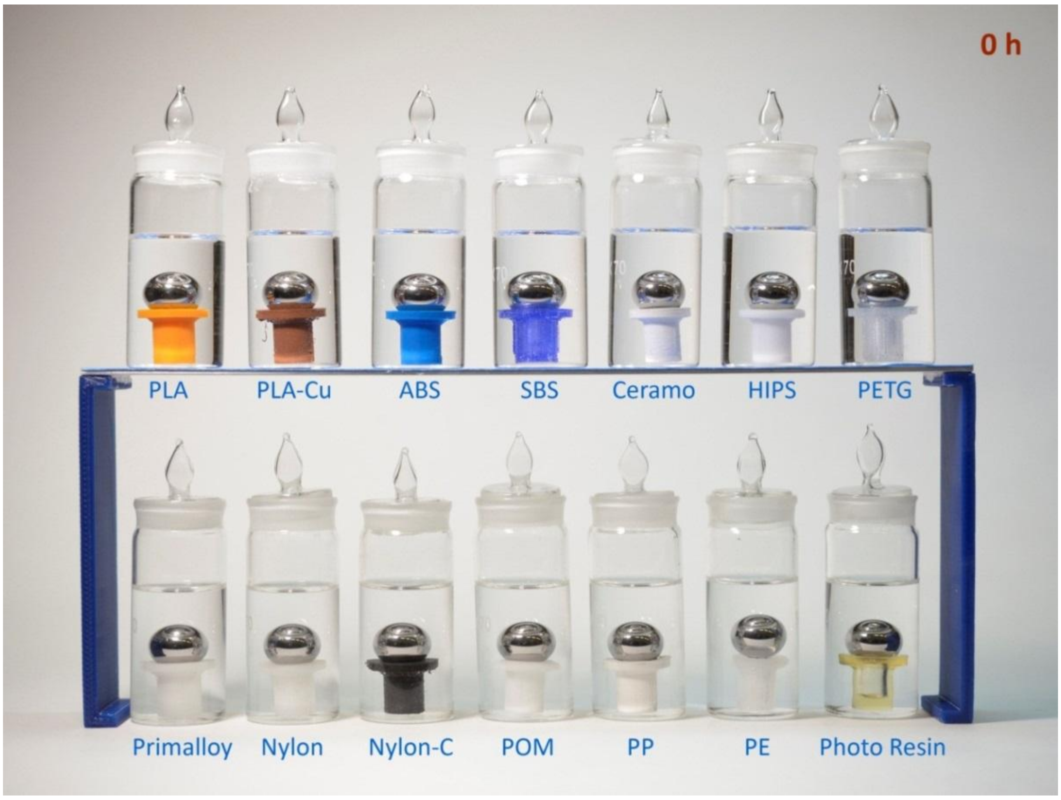

Figure S32. Snapshot of the experiment in ethanol in the beginning.

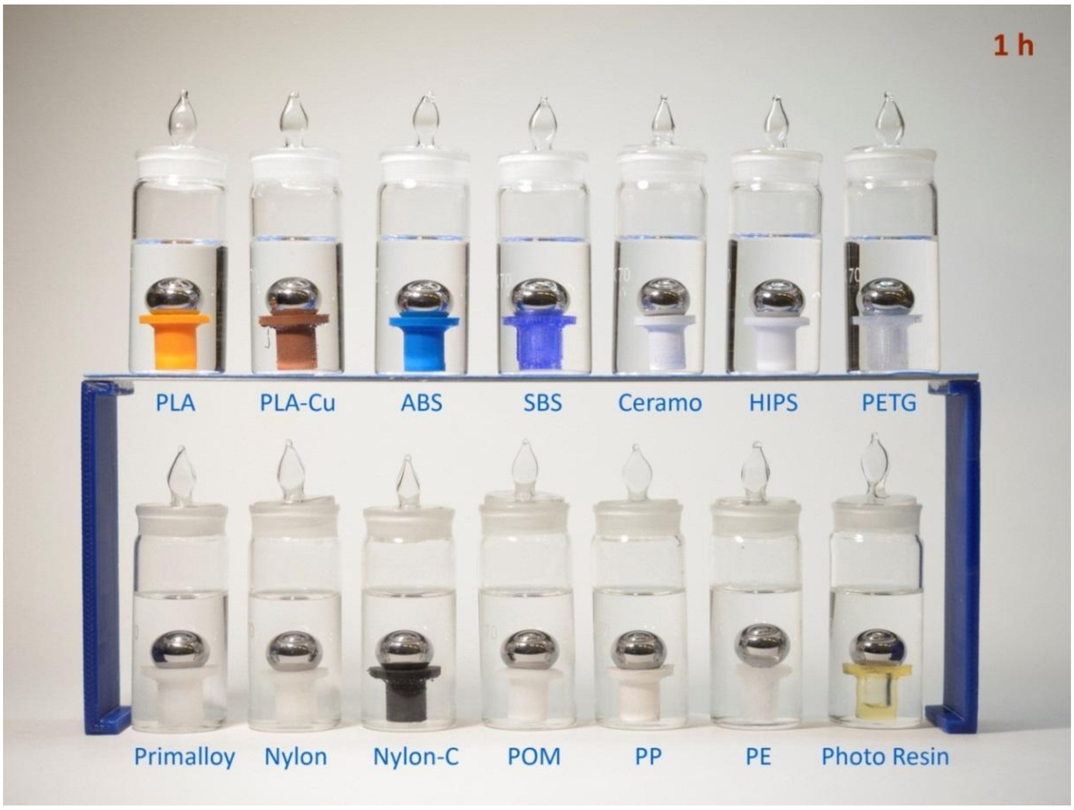

Figure S33. Snapshot of the experiment in ethanol after 1 h.

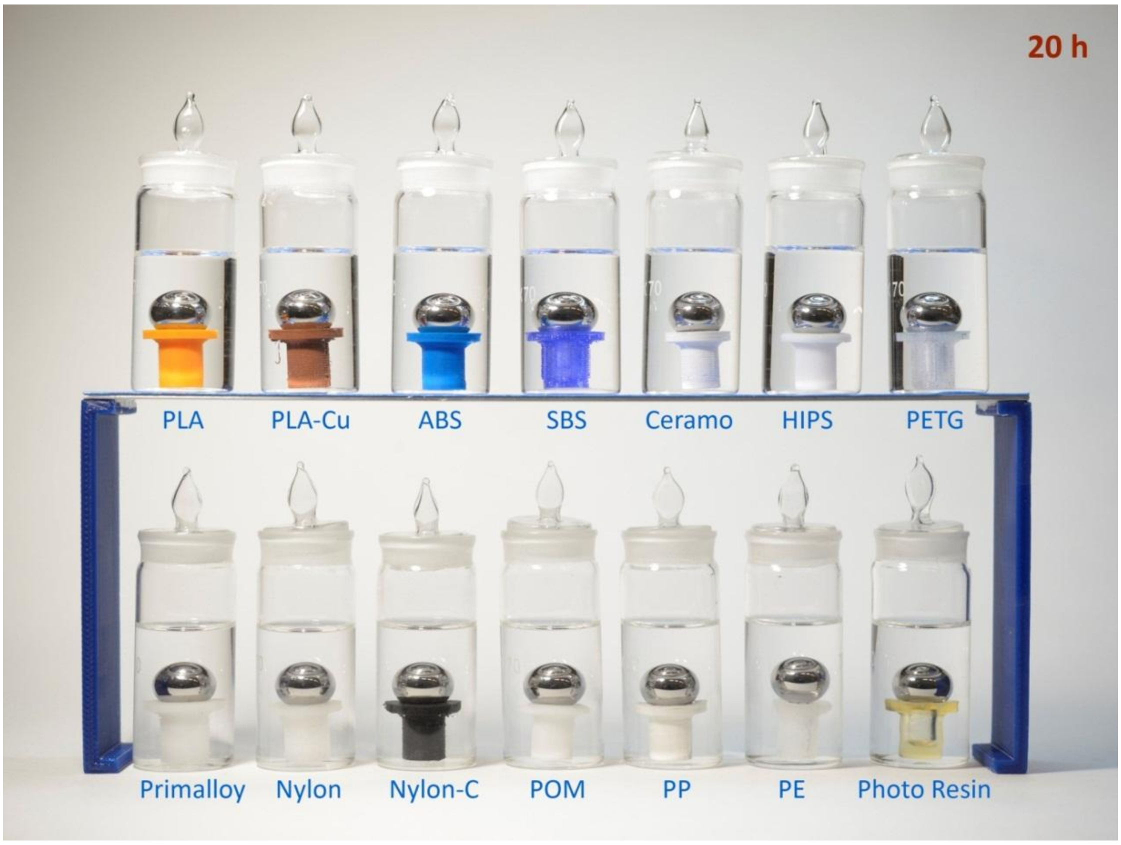

Figure S34. Snapshot of the experiment in ethanol after 20 h.

12. Experiments in media of water solution of acid (H_2SO_4 0.5M)

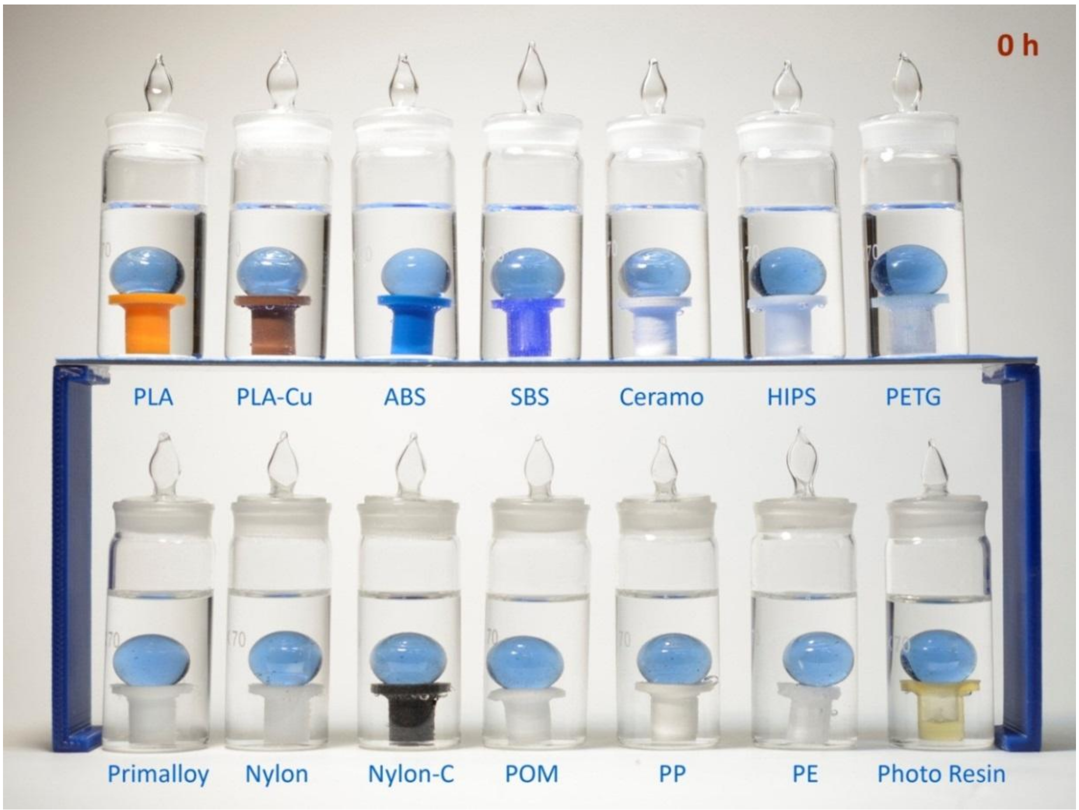

Figure S35. Snapshot of the experiment in water solution of H_2SO_4 (0.5 M) in the beginning.

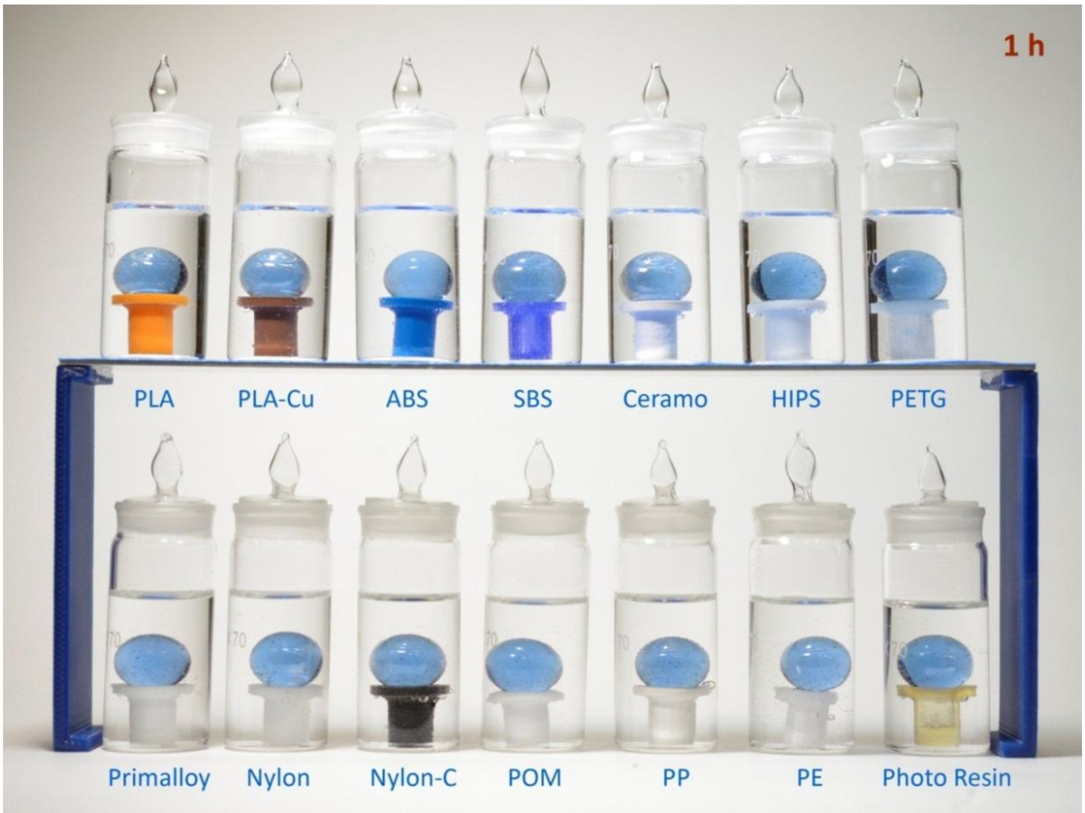

Figure S36. Snapshot of the experiment in water solution of H_2SO_4 (0.5 M) after 1 h.

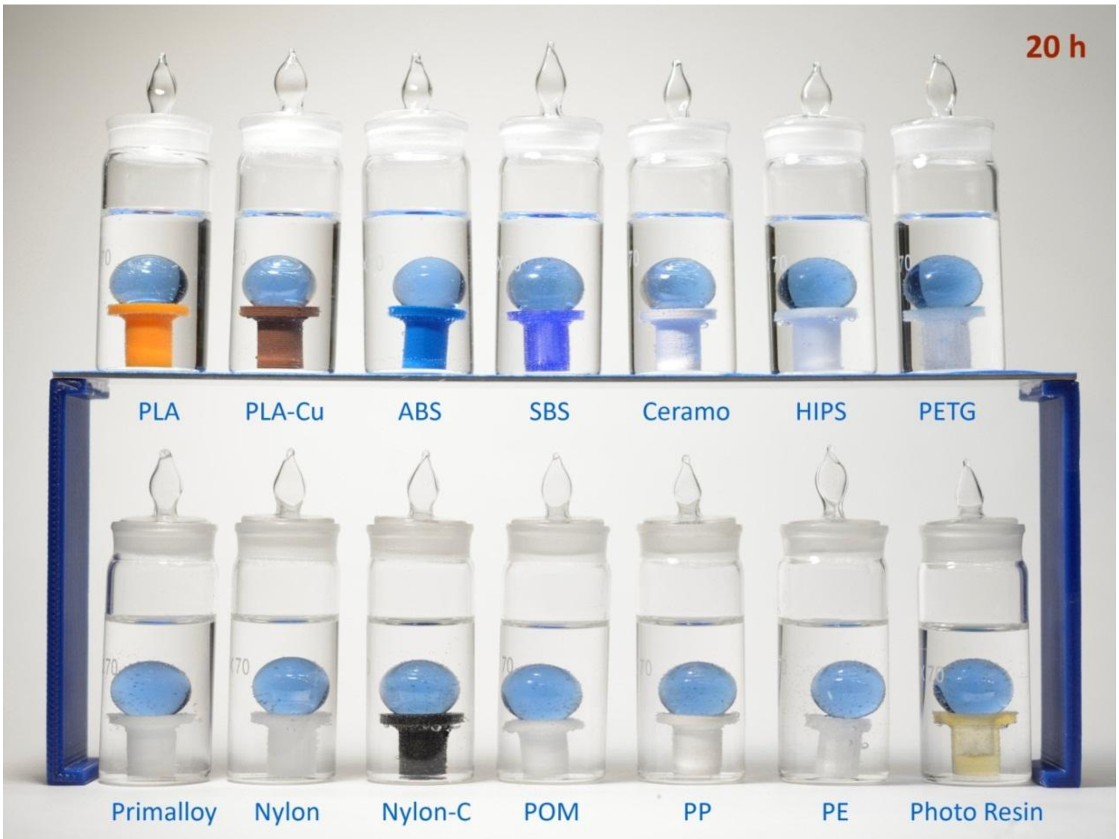

Figure S37. Snapshot of the experiment in water solution of H_2SO_4 (0.5 M) after 20 h.

13. Experiments in media of water solution of base (NaOH 1M)

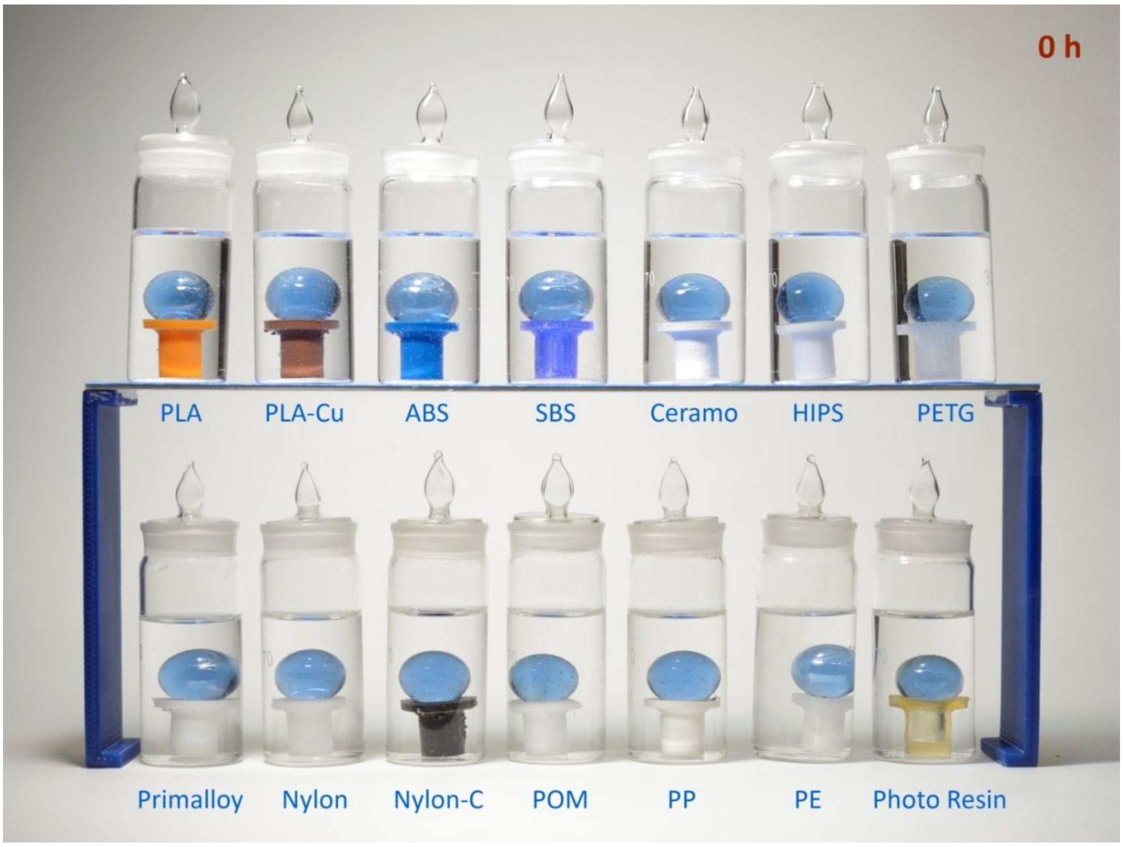

Figure S38. Snapshot of the experiment in water solution of NaOH (1M) in the beginning.

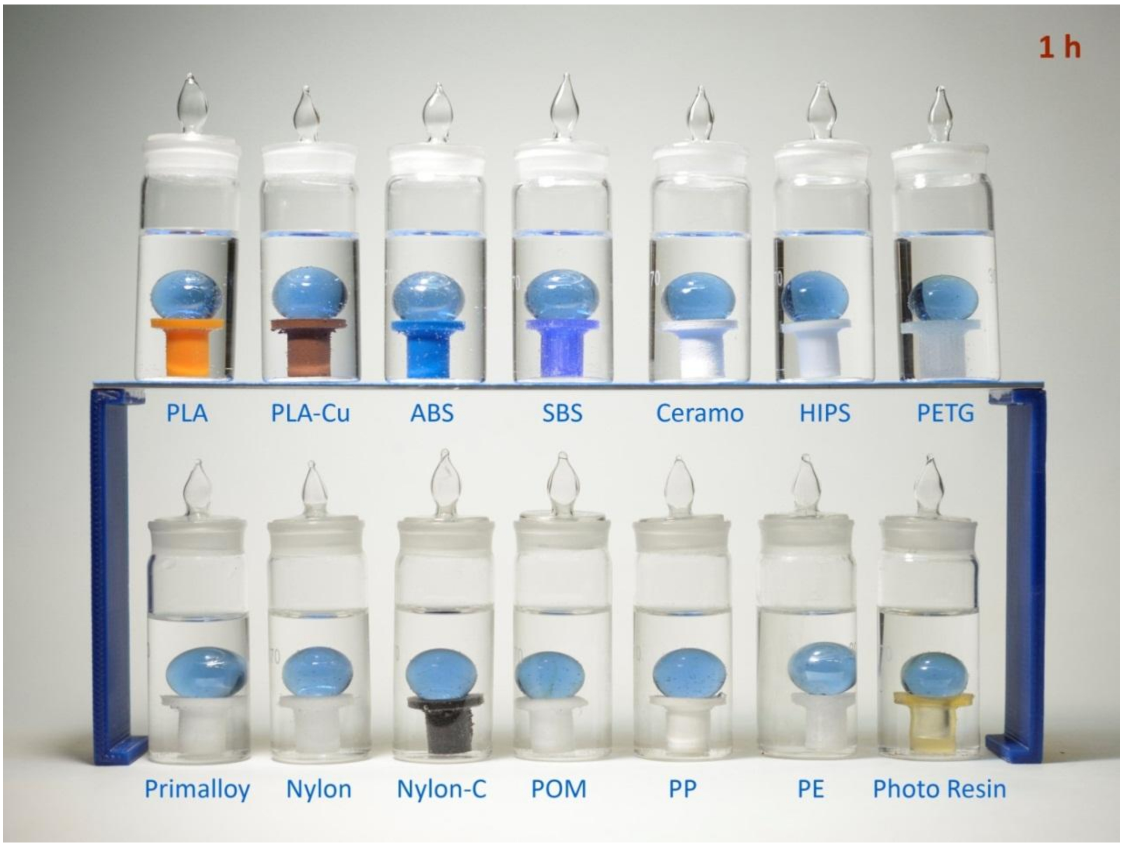

Figure S39. Snapshot of the experiment in water solution of NaOH (1M) after 1 h.

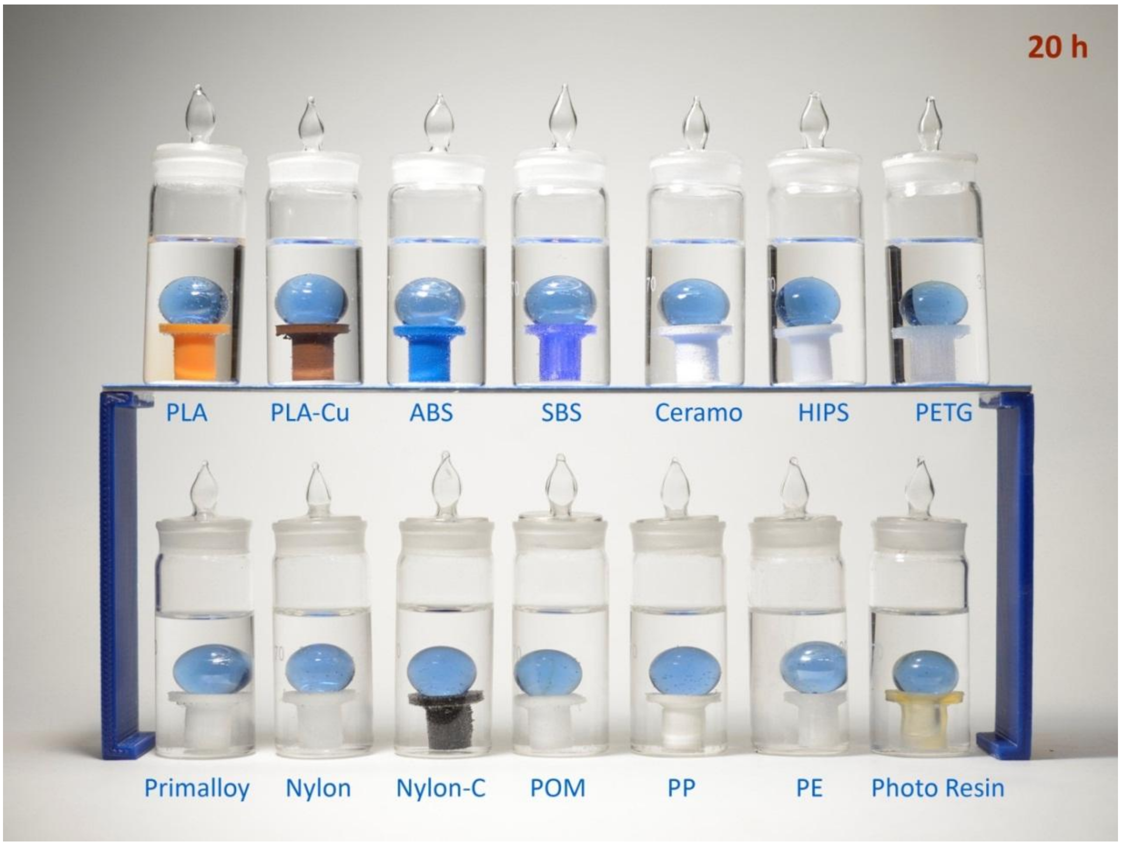

Figure S40. Snapshot of the experiment in water solution of NaOH (1M) after 20 h.

14. Experiments in water media

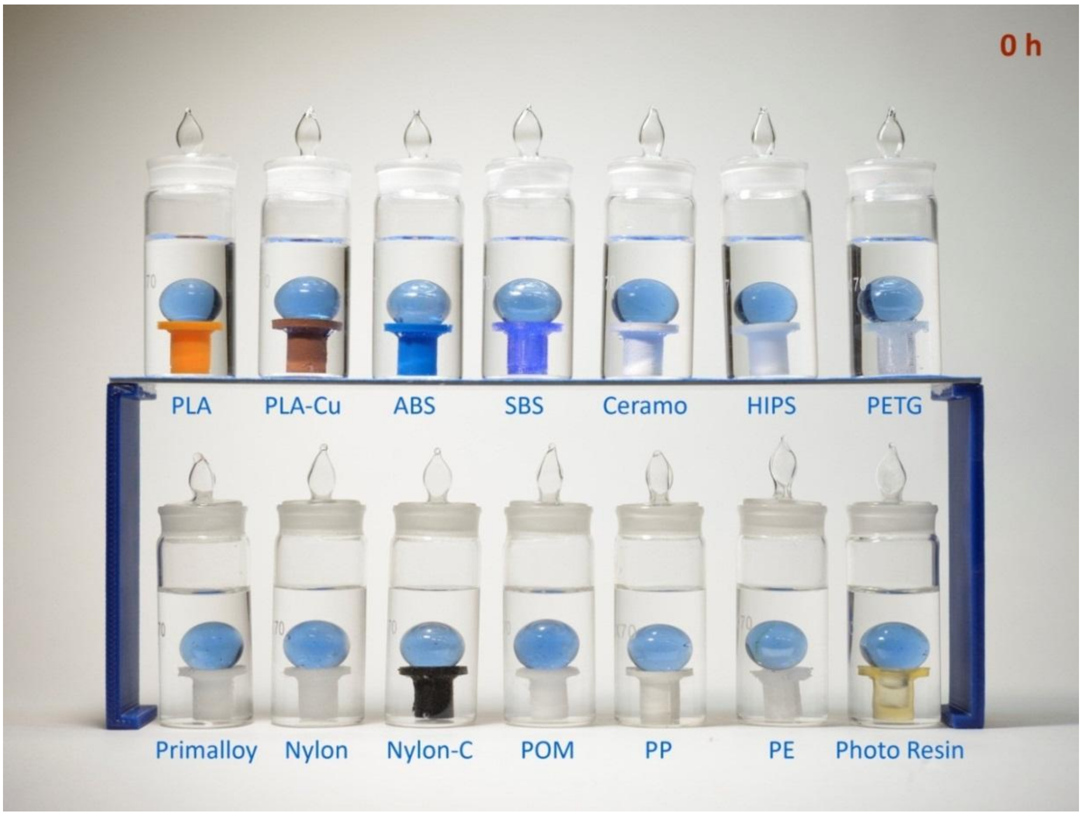

Figure S41. Snapshot of the experiment in water in the beginning.

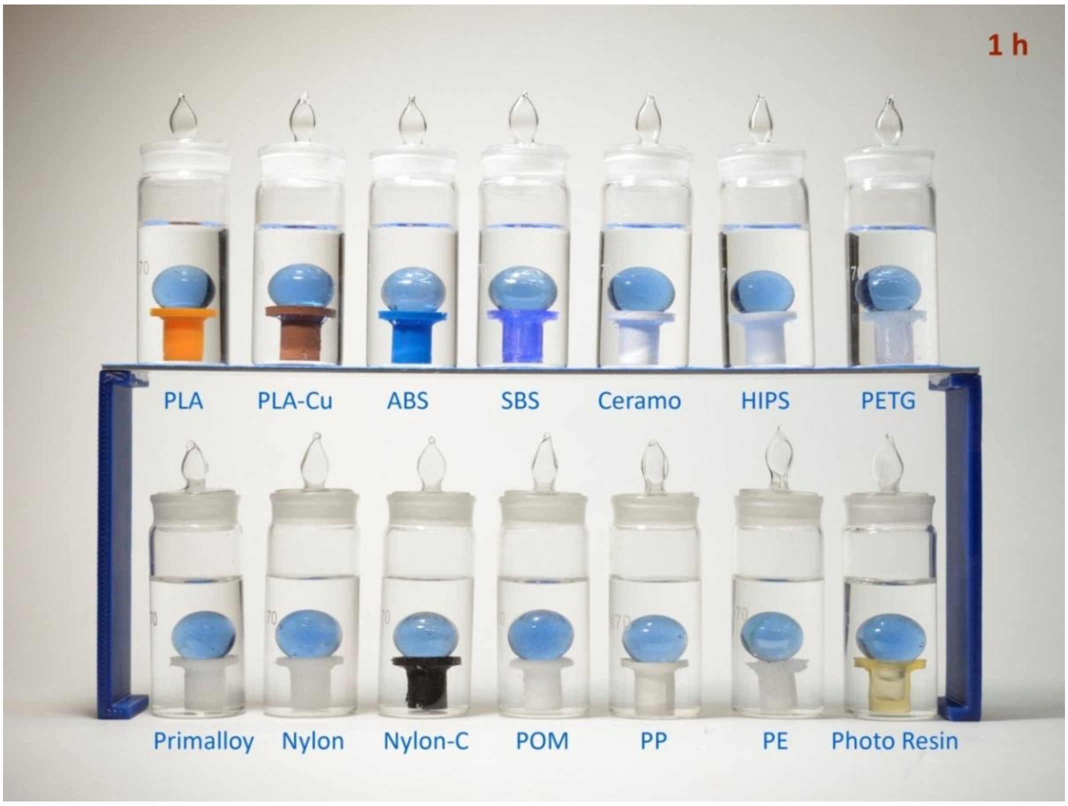

Figure S42. Snapshot of the experiment in water after 1 h.

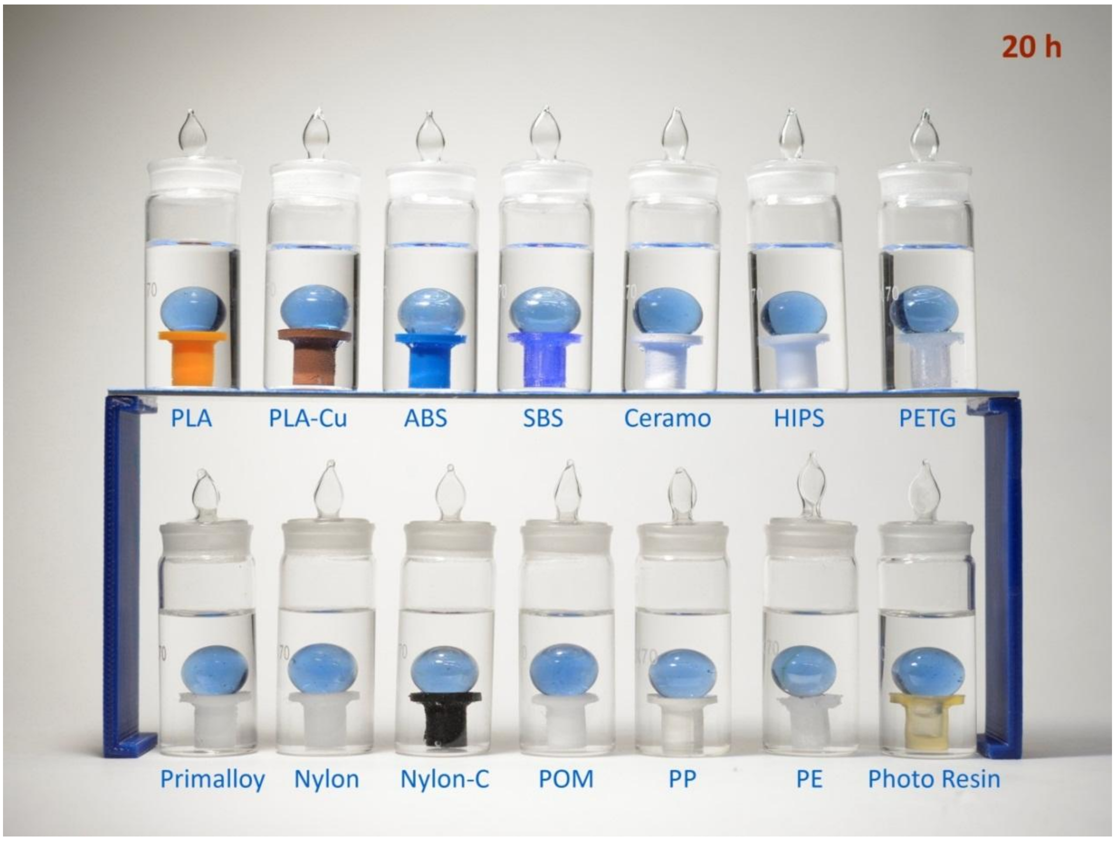

Figure S43. Snapshot of the experiment in water after 20 h.

15. Total table of FDM materials stability

Table S2. Qualitative analysis of stability of FDM parts made of different materials in organic and inorganic liquid media: (●) material is stable during experimental time i. e. shape of the part does not change; dissolution of outer layers of material does not occur; (●) material is not stable during experimental time, change of shape is observed, dissolution (DS), disintegration (DI), or/and delamination (DL) of the part occur; (●) material is moderately stable during experimental time: its swelling (SW) or slight dissolution of outer layers are observed, shape of the part does not change.

		PLA	PLA-Cu	ABS	SBS	Ceramo	HIPS	PETG	Primalloy	Nylon	Nylon-C	POM	PP	PE	Photo Resin
Water	1 h														
	20 h														
NaOH solution (1 M)	1 h														
	20 h														
H_2SO_4 solution (0.5 M)	1 h														
	20 h														
Ethanol	1 h														
	20 h														
Acetic acid	1 h		SW						SW						
	20 h	SW	SW		SW	SW			SW						SW
TEA	1 h				SW/DS		SW		SW				SW	SW	
	20 h		SW		SW/DS		SW		SW				SW	SW	
Ethyl Acetate	1 h	SW/DL	SW/DL	SW/DL	DL	DI	DS		SW						
	20 h	SW/DL	SW/DL	SW/DL	DL	DI	DS	DL	SW						
Toluene	1 h	SW/DL	SW/DL	SW/DL	DL	DI	DS		SW						
	20 h	SW/DL	SW/DL	SW/DL	DL	DI	DS		SW						
DMF	1 h	SW	SW	SW	SW/DS	DI	DS	SW	SW						
	20 h	SW	SW/DL	SW	DS	DI	DS	SW	SW						SW
Acetone	1 h	DL	DL	SW/DL		DI	DS								
	20 h	DL	DL	SW/DL	DS	DI	DS		SW						
THF	1 h	SW/DS	SW	SW	DS	DI	DS	SW	SW/DL				SW	SW	
	20 h	SW/DS	SW/DL	SW	DS	DI	DS	SW	SW/DL				SW	SW	SW
Methylene chloride	1 h	DS	DI	SW/DL	DL	DI	DS		SW						
	20 h	DS	DI	SW/DL	DL	DI	DS	DL	SW						

16. Experiment on ingredient outwashing from PLA-Cu in acetone media

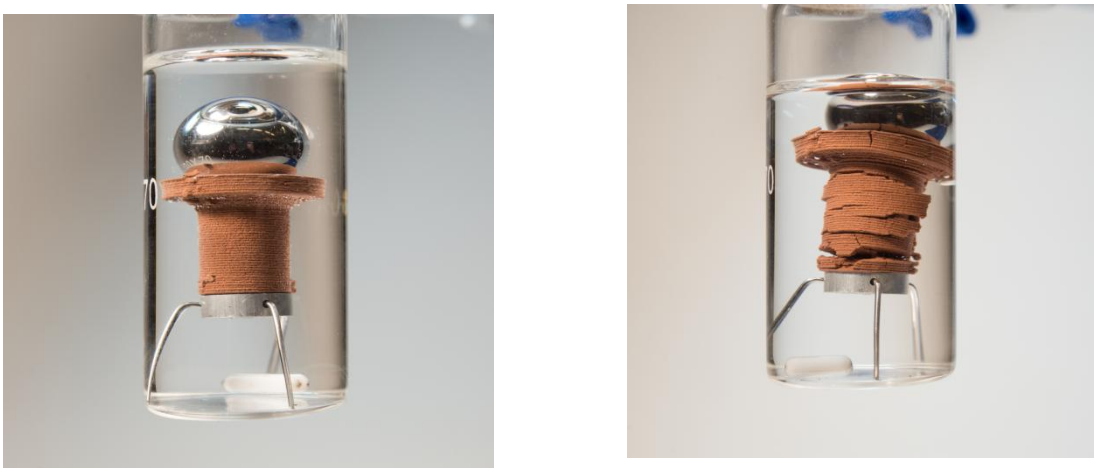

Figure S44. Snapshot of the experiment on ingredient outwashing from PLA-Cu part in acetone in the beginning and after 20 h.

17. Study of effect of Archimede’s buoyant force

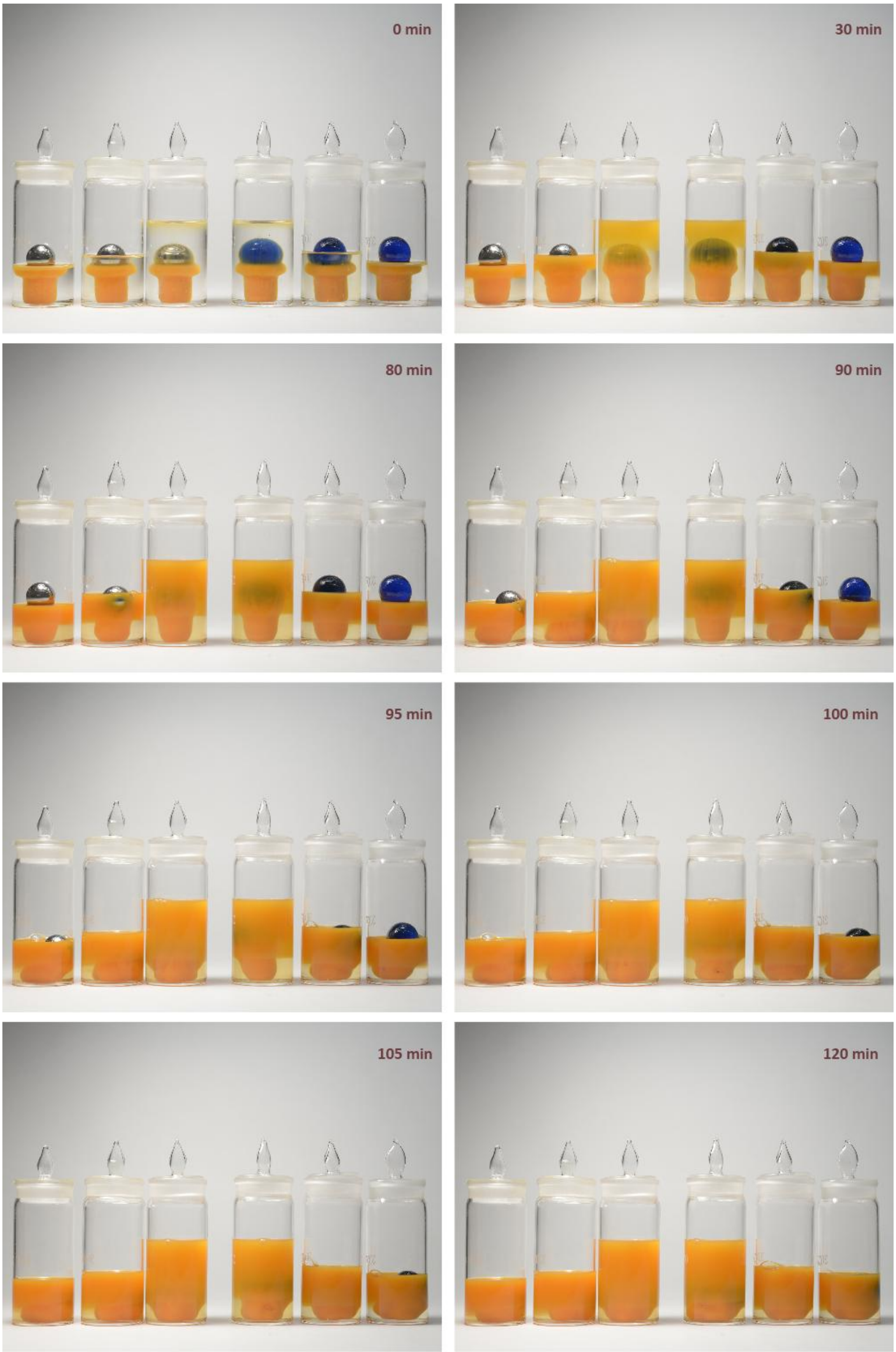

Figure S45. Destruction of FDM parts made of PLA with use of balls made of glass and stainless steel and different volume of DCM. Time of complete destruction of FDM part depends on molecular weight of polymer presented in filament. Composition of filaments of the same type purchased from different suppliers may vary.

18. Study of influence of indicator shape

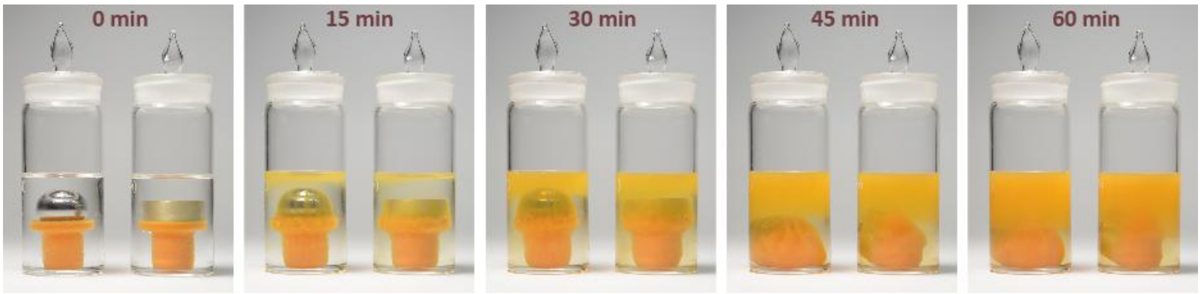

Figure S46. Destruction of FDM parts made of PLA with use ball and cylinder indicators of the same weight.

19. Influence of the extrusion multiplier on the structural stability of FDM parts in dichloromethane media

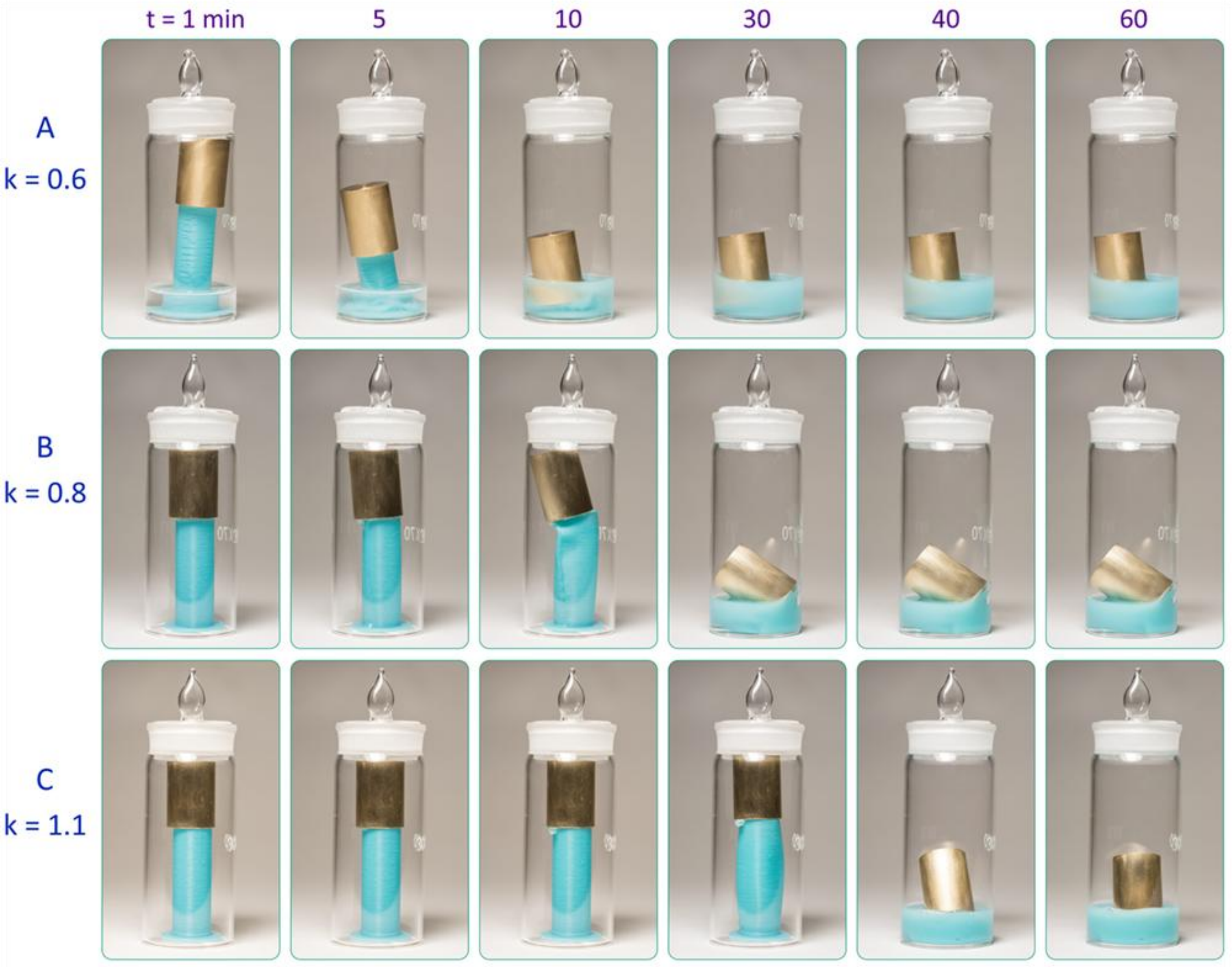

Figure S47. Destruction of FDM test tubes made of PLA with different extrusion multipliers (k) in DCM media.

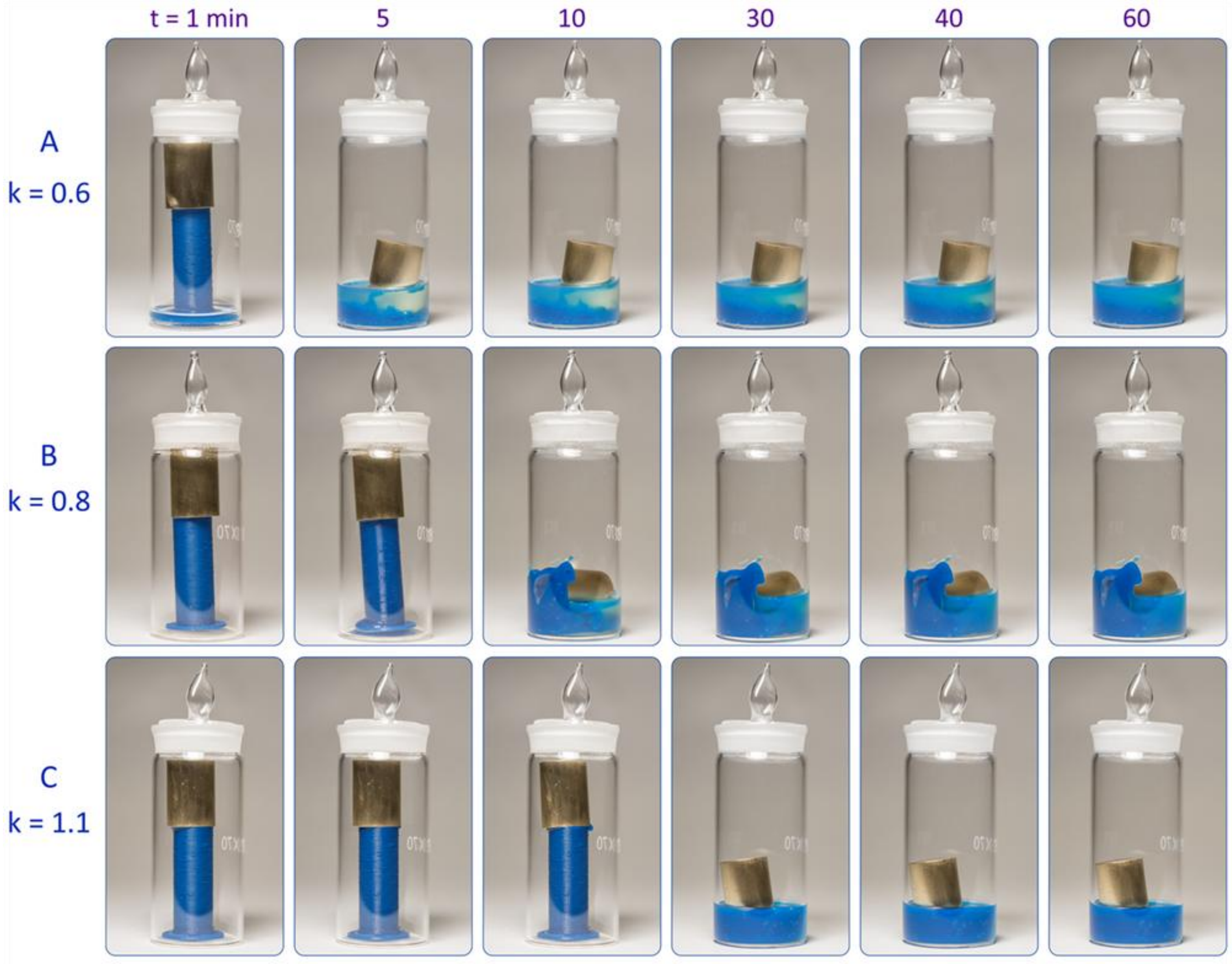

Figure S48. Destruction of FDM test tubes made of ABS with different extrusion multipliers (k) in DCM media.

20. Comparison of resistance of the extruded part and FDM parts

Table S3. Weights of the FDM parts and extruded part; k is extrusion multiplier.

**Method**	**k**	**m, g**
FDM	0.8	0.19
	0.9	0.20
	1.0	0.21
	1.1	0.21
	1.2	0.21
	1.3	0.20
Extrusion	-	0.21

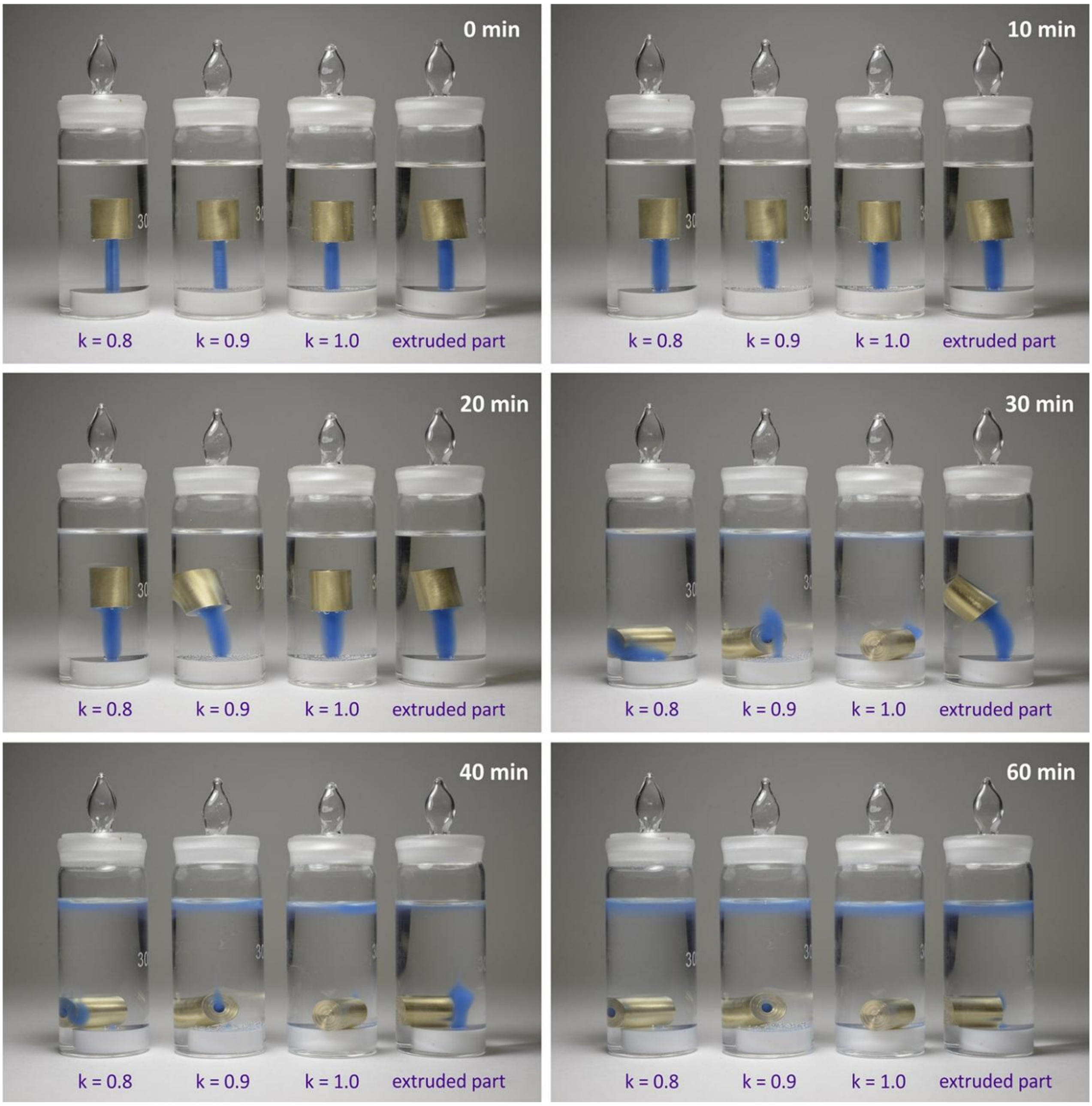

Figure S49. Destruction of the FDM parts made of PLA with different extrusion multipliers (k = 0.8 – 1.0) and extruded PLA-part in DCM media (see Supplementary_Movie_S1).

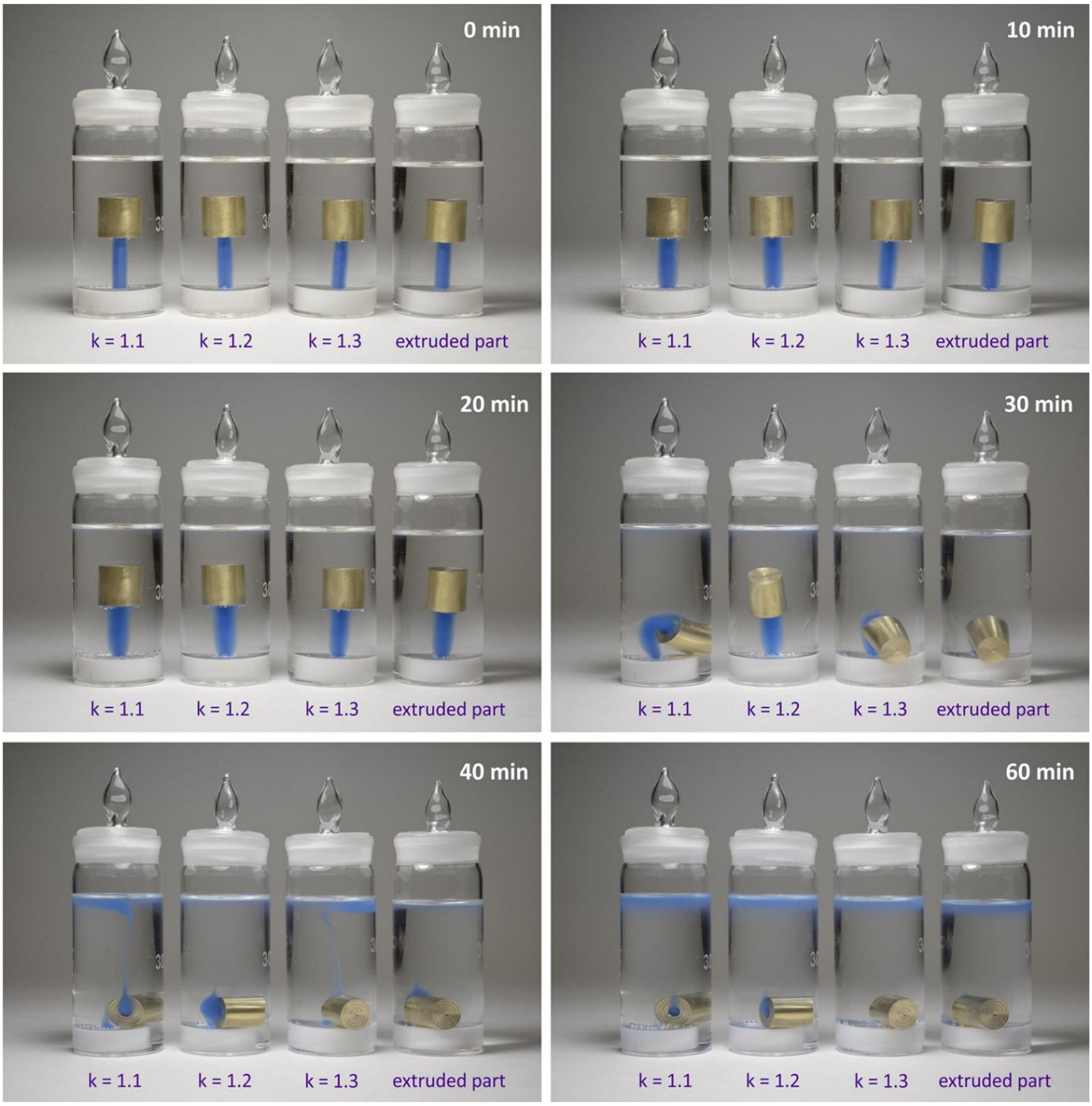

Figure S50. Destruction of the FDM parts made of PLA with different extrusion multipliers (k = 1.1 – 1.3) and extruded PLA-part in DCM media (see Supplementary_Movie_S2).

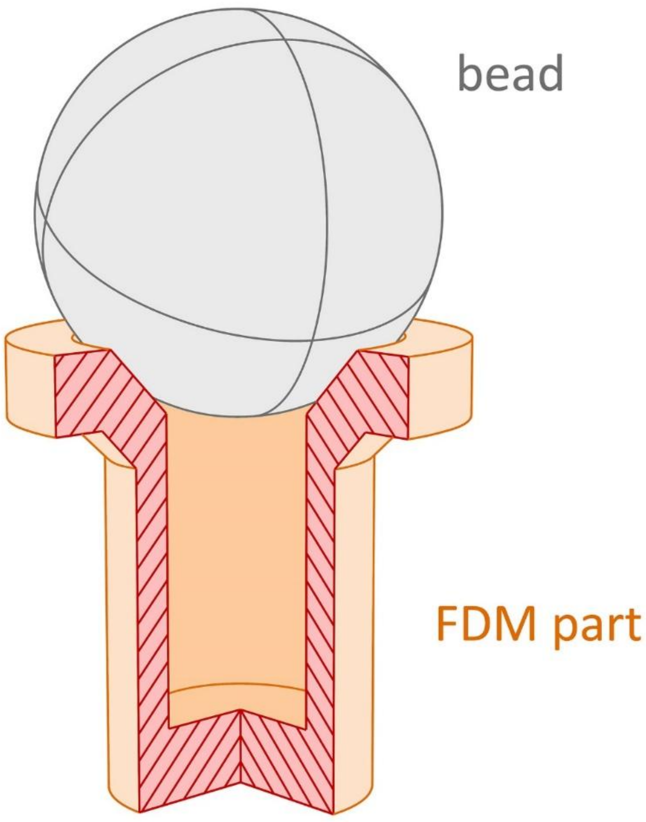

Figure S51. 3D model of test part with bead loading used for experiments in different solvents.
